# Unusual conservation of mitochondrial gene order in *Crassostrea *oysters: evidence for recent speciation in Asia

**DOI:** 10.1186/1471-2148-10-394

**Published:** 2010-12-28

**Authors:** Jianfeng Ren, Xiao Liu, Feng Jiang, Ximing Guo, Bin Liu

**Affiliations:** 1 Key Laboratory of Experimental Marine Biology, Institute of Oceanology, Chinese Academy of Sciences, Qingdao 266071, China; 2 Center of Systematic Genomics, Xinjiang Institute of Ecology and Geography, Chinese Academy of Sciences, Urumqi 830011, China; 3 Haskin Shellfish Research Laboratory, Institute of Marine and Coastal Sciences, Rutgers University, NJ 08349, USA; 4 Current Address: Institute of Genetics and Development Biology, Chinese Academy of Sciences, Beijing 100101, China; 5 Current Address: Institute of Zoology, Chinese Academy of Sciences, Beijing 100101, China

## Abstract

**Background:**

Oysters are morphologically plastic and hence difficult subjects for taxonomic and evolutionary studies. It is long been suspected, based on the extraordinary species diversity observed, that Asia Pacific is the epicenter of oyster speciation. To understand the species diversity and its evolutionary history, we collected five *Crassostrea *species from Asia and sequenced their complete mitochondrial (mt) genomes in addition to two newly released Asian oysters (*C. iredalei *and *Saccostrea mordax*) for a comprehensive analysis.

**Results:**

The six Asian *Crassostrea *mt genomes ranged from 18,226 to 22,446 bp in size, and all coded for 39 genes (12 proteins, 2 rRNAs and 25 tRNAs) on the same strand. Their genomes contained a split of the *rrnL *gene and duplication of *trnM*, *trnK *and *trnQ *genes. They shared the same gene order that differed from an Atlantic sister species by as many as nine tRNA changes (6 transpositions and 3 duplications) and even differed significantly from *S. mordax *in protein-coding genes. Phylogenetic analysis indicates that the six Asian *Crassostrea *species emerged between 3 and 43 Myr ago, while the Atlantic species evolved 83 Myr ago.

**Conclusions:**

The complete conservation of gene order in the six Asian *Crassostrea *species over 43 Myr is highly unusual given the remarkable rate of rearrangements in their sister species and other bivalves. It provides strong evidence for the recent speciation of the six *Crassostrea *species in Asia. It further indicates that changes in mt gene order may not be strictly a function of time but subject to other constraints that are presently not well understood.

## Background

Mitochondrial (mt) DNA is widely used for phylogenetic analysis because of its unique architecture, inheritance and small size. Metazoan mtDNA is nearly always a circular molecule except for some cnidarians [1]. It contains the same 37 genes, specifying 13 proteins of the respiratory chain [cytochrome c oxidase subunits I-III (*cox1-cox3*), apocytochrome b (*cob*), ATP synthase subunits 6 and 8 (*atp6 *and *atp8*), and NADH dehydrogenase subunits 1-6 and 4L (*nad1-6, nad4L*)], 2 ribosomal RNAs and 22 transfer RNAs. Although there are exceptions, most mtDNAs range in size from 14 to 17 kb. Typically, there are few intergenic nucleotides except for a single large non-coding region generally thought to contain elements that control the initiation of replication and transcription [2]. Size variation in mtDNA is usually due to the different length of the non-coding regions. Gene order is generally conserved in most metazoan taxa although some groups show considerable variation.

Variations in both mtDNA sequence and gene order have been used for phylogenetic analysis. Because mtDNA is fast evolving and nucleotide mutations may return to an early state, mtDNA sequences may not allow deep phylogenetic reconstruction. Gene order, on the other hand, has very small probability of back-mutation and may be particularly useful for high level phylogenetic analysis. Although the mechanism of mtDNA rearrangement is poorly understood, mt gene order has been increasingly used for phylogenetic studies [3-6].

As the second species-rich phylum of the animal kingdom after Arthropoda, Mollusca exhibits tremendous variation in their mt genomes. Seven bivalve lineages (Mytilidae, Unionidae, Margaritiferidae, Hyriidae, Donacidae, Solenidae, and Veneridae) have been found to have an unusual mode of inheritance for mtDNA, termed doubly uniparental inheritance (DUI) [7]. Some pulmonate gastropods have unusual tRNAs lacking the T-stem or the D-stem, similar to nematode mt tRNAs [8]. The *atp6 *and *atp8 *genes are separated in the scaphopods and two groups of gastropods (Patellogastropoda and Heterobranchia). In addition, several mt genes are duplicated in cephalopods *Watasenia scintillans *and *Todarodes pacificus *[9]. Unlike the general conservation in gene order in most other metazoan groups, most molluscan mt genomes reported so far contain considerable rearrangements especially in Bivalvia and Scaphopoda [10]. At the same time, the phylogeny of molluscs is poorly studied. Phylogenetic relationships among major molluscan groups are not well understood. The species identity and classification of some most common molluscs remain questionable.

Oysters are bivalve molluscs widely distributed in world oceans. They are benthic, sessile filter-feeders with important roles in estuary ecology. Some species support major fishery and aquaculture industries worldwide. Despite the abundance, ecological and economic significance of oysters, we know little about their species diversity and evolutionary history. Classification of oysters remains a challenge partly due to the lack of well-defined morphological characters. Shell morphology, the main character used in oyster classification, is known to be plastic and subject to environmental variation. Much of the oyster classification to date is based on shell characteristics and has resulted in considerable errors and confusion. The difficulty of oyster classification is particularly pronounced in China and other parts of Asia where a large number of species are sympatric [11,12]. About thirty species have been recorded along the coast of China [13]. The presence of a large number of oyster species has led many to believe that Asia Pacific is the epicenter of oyster speciation, but the inability to reliably identifying them has hindered our understanding of oyster evolution. Among the extent oysters, five *Crassostrea *oysters, namely *C. gigas*, *C. angulata*, *C. sikamea*, *C. hongkongensis *and *C. ariakensis *are commonly found in China and other parts of Asia and yet, they have been difficult to identify by shell morphology alone. Recently, some confusion in the oyster identification have been resolved using DNA sequence data [12,14-20], and the five *Crassostrea *oysters can be reliably identified [21]. Still, we know little about the evolutionary history of the *Crassostrea *species and how they relate to each other and the other oysters. For example, it is not clear whether *C. angulata *should be considered as an independent species and which species is the closest relative of the newly described *C. hongkongensis*. Most of the phylogenetic analyses so far are based on short DNA fragments, yielding variable results [12,16,22].

The ability to sequence and compare whole mt genomes provides a new impetus for phylogenetic analysis of oysters and other molluscs. Complete mt genome sequences have been obtained for two *Crassostrea *oysters from Asia (*C. gigas *and *C. hongkongensis*) and one species from the Atlantic (*C. virginica*) [23-25]. Comparative analysis shows that the two Asian species share the same gene order that is very different from the Atlantic species [24,25]. To determine if gene order is conserved in other Asian species and to understand the evolution of *Crassostrea *oysters, we sequenced the mt genomes of four *Crassostrea *oysters from Asia plus one that was previously published [25], obtained newly released mt genomes of two Asian oysters (*C. iredalei *and *Saccostrea mordax*), and compared them with other molluscan genomes. Here we report the first estimates of divergence time among *Crassostrea *species based on complete mt sequences and the complete conservation in gene order among the six Asian *Crassostrea *species. The complete conservation of gene order is highly unusual considering the tremendous rearrangement of mtDNA in most marine bivalves. It provides strong evidence for recent emergence of the six *Crassostrea *species in Asia. Our analysis also suggests that rearrangement of the mt genome may not be strictly a function of time but subject to some other constraints that are presently not well understood.

## Results and Discussion

### Genome composition

**Figure 1:**
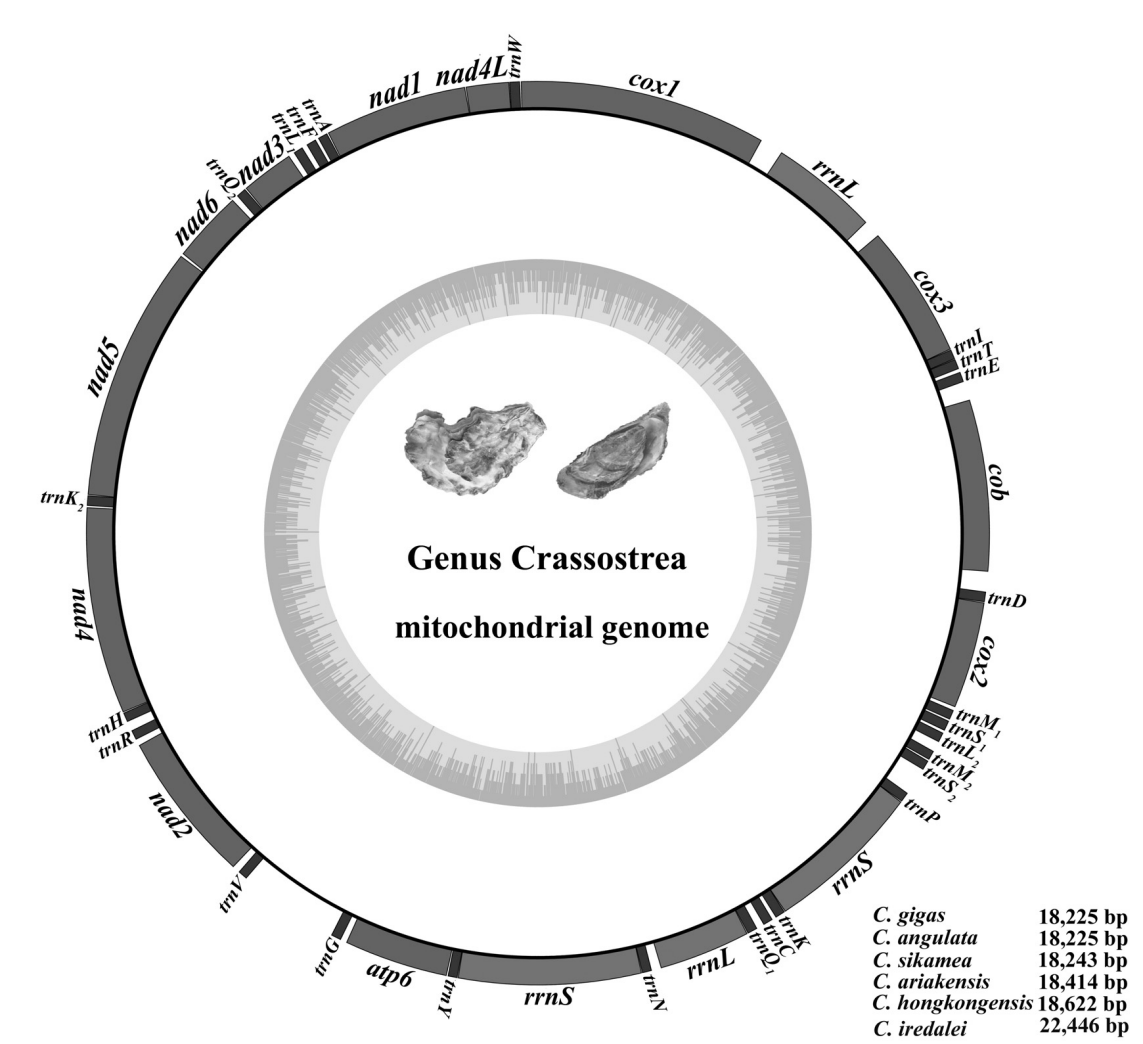
**Mitochondrial genome map of Asian *Crassostrea *oysters**. All 39 genes are coded on the same DNA strand. Genes for proteins and rRNAs are shown with standard abbreviations. Genes for tRNAs are designated by a single letter for the corresponding amino acid with two leucine tRNAs and two serine tRNAs differentiated by numeral. The two tRNA duplications of methionine, lysine and glutamine are named *trnM1, trnM2*, *trnK1*, *trnK2*, *trnQ1 *and *trnQ2*, respectively. The length of five mt genomes is indicated at the lower right-hand corner.

Genome composition and organization of the six Asian *Crassostrea *oysters is summarized in Figure 1 and additional file 1: Table S1. Organization of the American oyster *C. virginica*, a sister species from the Atlantic Ocean, is listed for comparative analysis (additional file 1: Table S1). The complete mt genomes of *C. gigas*, *C. angulata*, *C. sikamea*, *C. hongkongensis *and *C. ariakensis *are 18,225 bp, 18,225 bp, 18,243 bp, 18,622 bp and 18,414 bp in length, respectively [24,25]. These sequences have been deposited in GenBank under the accession number EU672831, NC_012648-NC_012650, NC_011518. The American oyster *C. virginica *mtDNA is 17,244 bp in length. The mt genome of *S. mordax *is 16,532 bp in length. However, the mt genome of *C. iredalei*, 22,446 bp in length, is obviously longer than all other oysters [54]. The size of molluscan mt genomes varies dramatically, ranging from 13,670 bp in the snail *Biomphalaria glabrata *to 40,725 bp in the sea scallop *Placopecten magellanicus *[41,42]. The six Asian *Crassostrea *oyster genomes encode 39 genes including 12 protein-coding genes, 2 rRNAs and 25 tRNAs on the same strand (Figure 1). In contrast to the typical animal mt genomes, they lack one protein-coding gene *atp8 *and have duplications of three tRNAs: *trnM*, *trnK *and *trnQ*. Another unique character is that *rrnL *gene is split into two segments (also in *C. virginica *and *S. mordax*) and *rrnS *has a nearly identical duplication, which has never been reported in other animal mt genomes [23]. Among the seven *Crassostrea *oysters, the lowest A+T content is 61.1% in *C. virginica*, while the highest A+T content is 65.3% in *C. hongkongensis*. In the other five species, the A+T content varies from 62.9% in *C. ariakensis *to 64.5% in *C. iredalei *[54] (Table 1). The highest A+T content of *C. hongkongensis *is mainly caused by the increased total length and high A+T content of the non-coding region. The length of coding regions in the Asian-Pacific oyster genomes is similar and larger than that in *C. virginica*, because of duplications of two tRNAs (*trnK *and *trnQ*) and one *rrnS*.

**Table 1:** Genomic characteristics of bivalve mtDNAs

Species	GenBank Accession Number	Positive strand		Protein-coding gene					*rrnL gene*		*rrnS gene*		tRNA		Largest NCR		Reference
			
		Length bp	A+T%	No. of AA	A+T%				Length bp	A+T%	Length bp	A+T%	Length bp	A+T%	Length bp	A+T%	
														
					Total	**1^st ^cdn pos**.	**2nd cdn pos**.	**3rd cdn pos**.									
*Lampsilis ornata*	NC_005335	16,060	62.4	3,710	61.6	56.5	61.6	67.1	1,315	62.8	846	60.5	1,368	64.6	283	64.7	[10]
*Inversidens japanensis*	AB055625	16,826	57.2	3,647	56.6	53.6	59.4	56.9	1,304	58.3	845	56.6	1,416	60.5	1,196	57.5	Okazaki et al, unpublished
*Hiatella arctica*	NC_008451	18,244	66.4	3,968	65.8	61.8	63.4	72.2	1,447	67.0	901	63.0	1,472	67.0	614	66.1	[48]
*Acanthocardia tuberculata*	NC_008452	16,104	59.9	3,636	59.4	55.8	61.0	61.4	1,213	65.2	824	59.5	1,489	57.8	1,103	59.1	[48]
*Venerupis philippinarum*	NC_003354	22,676	69.7	4,211	68.5	62.2	63.0	68.1	1,408	72.8	1,249	70.6	1,459	69.2	2,183	68.2	Okazaki et al, unpublished
*Meretrix petechialis*	NC_012767	19,567	68.3	4,014	66.9	60.5	64.4	75.6	1,581	71.0	1,187	69.4	1,458	68.4	1,634	69.4	[50]
*Mytilus galloprovincialis*	NC_006886	16,744	61.8	3,733	60.5	56.0	62.1	63.3	1,244	65.6	947	64.2	1,517	66.7	1,157	60.2	[46]
*Mytilus edulis*	NC_006161	16,740	61.8	3,681	60.5	56.0	62.0	63.6	1,244	65.3	945	64.1	1,517	66.6	1,158	61.0	[51]
*Mytilus trossulus*	NC_007687	18,652	61.5	3,716	59.5	54.9	62.1	61.6	1,244	66.8	948	63.6	1,584	67.1	1,561	63.9	[47]
*Placopecten magellanicus*	NC_007234	32,115	55.7	3,742	55.7	54.3	58.1	54.6	1,387	58.0	970	52.9	2,294	50.1	3,112	60.4	[42]
*Mizuhopecten yessoensis*	NC_009081	20,414	55.2	3,763	55.5	53.3	58.2	54.9	1,424	57.8	961	50.6	1,079	49.4	1,528	59.9	Sato et al, unpublished
*Argopecten irradians*	DQ665851	16,211	57.3	3,681	57.0	52.7	58.5	59.6	1,292	59.5	904	56.8	1,365	52.5	1,038	63.6	[52]
*Chlamys farreri*	EF473269	20,789	58.7	3,737	58.9	55.2	58.4	63.1	1,479	58.3	953	52.4	1,415	50.5	3,859	63.7	[52]
*Crassostrea virginica*	NC_007175	17,244	61.1	3,696	60.1	55.0	60.5	64.7	1,469	63.7	989	57.2	1,567	61.8	832	65.7	[23]
*Crassostrea gigas*	EU672831	18,225	63.4	3,718	62.8	57.1	60.4	71.0	1,314	65.1	2,242	60.1	1,693	63.4	645	69.8	This study
*Crassostrea angulata*	NC_012648	18,225	63.1	3,717	62.4	57.0	60.3	69.8	1,315	65.2	2,244	60.1	1,691	63.3	643	69.5	This study
*Crassostrea sikamea*	NC_012649	18,243	63.4	3,717	62.7	57.1	60.1	70.9	1,314	64.8	2,232	60.2	1,694	63.4	655	71.5	This study
*Crassostrea hongkongensis*	NC_011518	18,622	65.3	3,701	64.5	58.3	60.0	75.3	1,317	64.8	2,264	61.6	1,704	64.6	608	77.6	[25]
*Crassostrea ariakensis*	NC_012650	18,414	62.9	3,699	62.5	57.0	60.3	70.3	1,318	63.7	2,253	59.4	1,698	63.1	716	67.4	This study

### Gene arrangement

**Figure 2:**
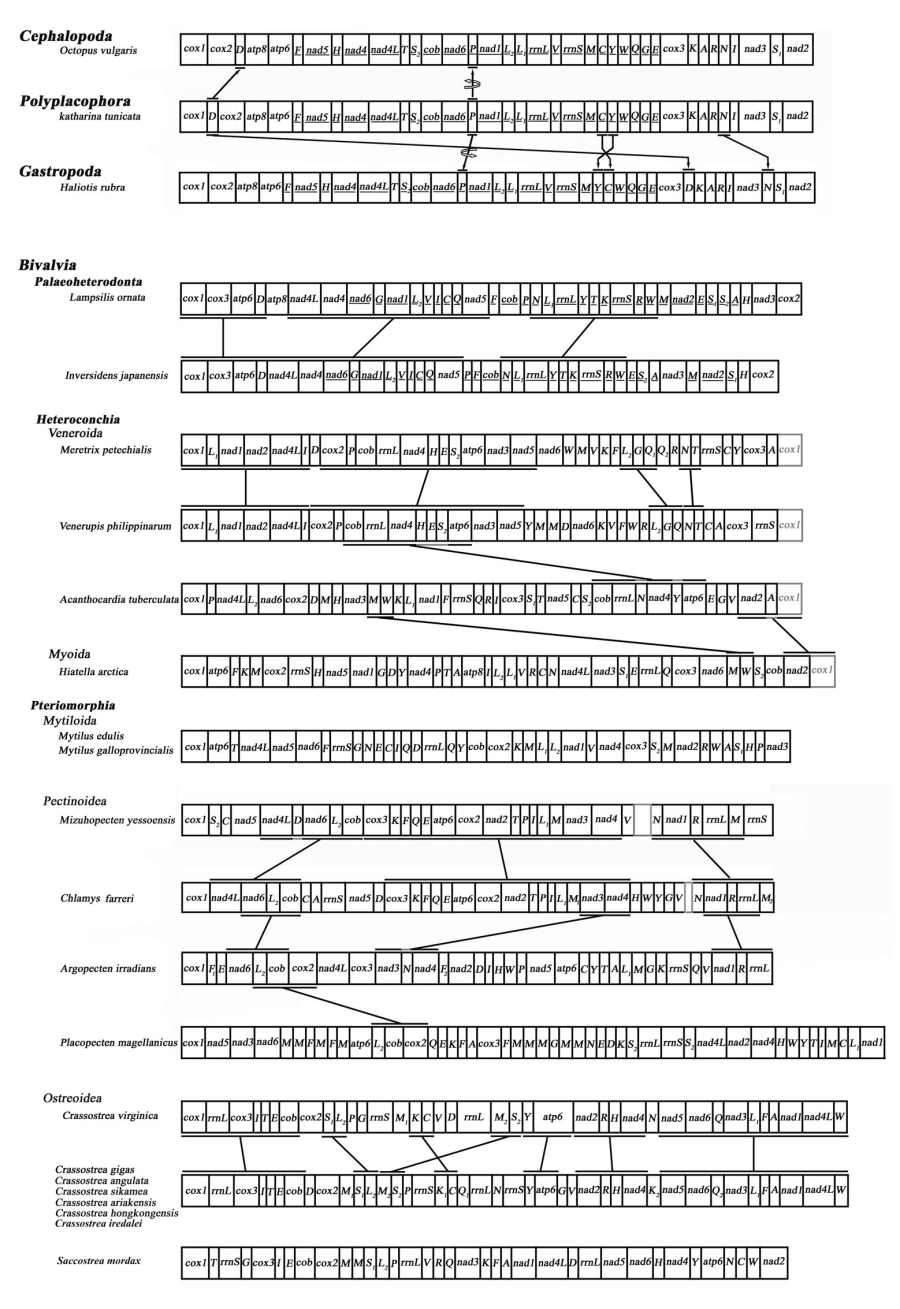
**Mitochondrial genome rearrangements in bivalves and representatives from other molluscan classes**. All genes are transcribed from left-to-right except those indicated by underlining, which are transcribed from right to left. The bars show identical gene blocks. The arrows indicate gene translocation and the circling arrows indicate gene inversion. The grey blank boxes represent the uncompleted sequences. The non-coding regions are not presented and gene segments are not drawn to scale.

Molluscs, especially bivalves, display an extraordinary amount of variation in gene arrangement. Gene order of selected molluscs was presented and compared with that of oysters in Figure 2. The black chiton *Katharina tunicata *is the only sequenced representative of the Polyplacophora, an early diverged class of the Mollusca [43]. Its gene order may represent the pleisomorphic gene arrangement in Mollusca. At high taxonomic levels, the gene order tends to be conserved across polyplacophoran, cephalopods and gastropods. The gene order of *K. tunicata *differs from that of the common octopus *Octopus vulgaris *by merely the inversion of *trnP *and translocation of *trnD*. Gene order of other cephalopods resembles that of *O. vulgaris *with some translocations of tRNA or the switch of large gene blocks [9]. Additionally, the gene arrangement of *K. tunicata *differs from that of the blacklip abalone *Haliotis rubra *merely by the inversion of *trnP *plus transposition of four tRNAs.

In comparison, however, mt genomes of Bivalvia show tremendous gene rearrangements. To date, all bivalves whose mt genomes are available belong to three subclasses: Palaeoheterodonta, Heterodonta and Pteriomorphia. Gene order in freshwater mussel *Lampsilis ornata *(Palaeoheterodonta: Unionoida) is nearly identical to that of it sister species *Inversidens japanensis *except for the translocation of several tRNAs, and protein-coding genes *nad2 *and *nad3 *(Figure 2) [10]. In addition, *L. ornata *genome contains the *atp8 *gene which is absent in *I. japanensis *(Data not shown). The remaining bivalves whose mt genomes have been sequenced are marine species, where the gene order is dramatically rearranged. Comparing the gene arrangement between *Meretrix petechialis *and *Venerupis philippinarum *(Heterodonta: Veneridae), they share four identical gene blocks: two large blocks *cox1-L1-nad1-nad2-nad4L-I *and *cox2-P-cob-rrnL-nad4-H-E-S2-atp6-nad3-nad5*, and two small blocks only containing tRNA genes. They share only one gene block *cob-rrnL-nad4-atp6 *with *Acanchocardia tuberculata *of the same order. Meanwhile, *A. tuberculata *and *Hiatella arctica *share only two small gene blocks *trnM-W *and *nad2-cox1*. Species sequenced in Pteriomorphia belong to three orders: Mytiloida, Ostreoida and Pectinoida. Few gene blocks are shared between any two pairs. Among the four scallops of the family Pectinidae (Pectinoida), gene arrangement of *Mizuhopecten yessoensis *closely resembles that of *Chlamys farreri*. They share three large gene blocks *nad4L-nad6-L_2_-cob*, *cox3-K-F-Q-E-atp6-cox2-nad2-T-P-I-L_1_-M-nad3-nad4 *and *N-nad1-R-rrnL-M*. But their gene order is very different from that of the other two scallops, despite being members of the same family. The two genomes of *C. farreri *and *Argopecten irradians *only show three small shared gene blocks *nad6-L2-cob*, *nad3-nad4 *and *nad1-R-rrnL*, while *A. irradians *and *P. magellanicus *only share one gene block *L_2_-cob-cox2*.

On the other hand, the mt gene order in the six Asian *Crassostrea *oysters is completely identical to each other (Figure 2). This is highly unusual considering the tremendous rearrangement observed in other groups of Bivalvia. Even within the same genus, comparison between the Asian *Crassostrea *oysters of the Pacific and the American oyster *C. virginica *of the Atlantic Ocean show that gene order has been drastically rearranged. Gene order of the protein-coding genes (PCGs) is the same, while several tRNAs has translocated after the divergence between the Asian and American species. The six Asian *Crassostrea *oysters have duplicated gene *trnK*, *trnQ *and *rrnS *compared with the American oyster. They differ from the Atlantic sister species by as many as nine tRNA changes (6 transpositions and 3 duplications), and furthermore, they differ significantly from *S. mordax *in PCGs. If the tRNA is not considered, they share three PCGs blocks *cox1-cox3-cob-cox2*, *nad3-nad1-nad4L*, *nad5-nad6 *and *atp6-nad2*. It is amazing that *Crassostrea *sister species from the two oceans have so much difference in gene order and close relatives even have more difference in gene order, while gene order is completely conserved in the six *Crassostrea *species from Asia. This raises the questions what causes rearrangements of mt genomes and how do they evolve over time. Additional studies of mt genomes of other oyster species would be interesting to see if this pattern should preserve, which may further our understanding of mt genome evolution.

### Protein-coding genes

Of the 13 typical PCGs (*cox1-cox3*, *nad1-nad6*, *nad4L*, *cob*, *atp6 *and *atp8*), twelve genes were determined and the *atp8 *gene was absent in all oyster genomes. All PCGs are encoded on and transcribed from the same strand. These features have been observed in all other marine bivalve genomes published so far except for *H. arctica *where the *atp8 *gene has been reported, and for two species of Palaeoheterodonta (*L. ornate *and *I. japanensis*) where genes are coded on both strands (Figure 2). Thus, coding genes on the same strand and missing *atp8 *gene are the most distinct features of marine bivalve mt genomes, though from a recent publication from Breton et al [57] this might only hold true for *Ostreoida *and a few other bivalves. The two Palaeoheterodonta species that use both strands for coding are the only known members of Bivalvia showing significant conservation in gene order with other molluscan classes that also use both strands for coding. We suspect that coding on both strands may be inhibitory to mt genome rearrangement, and marine bivalves show a tremendous amount of mt genome rearrangement because they only use one strand for coding. Rearranging a genome with dual-strand coding may be more complicated and cause more harm than rearranging a genome that codes on one strand.

Mt genomes often use a variety of non-standard initiation codons [44]. In some cases, identification of the very clear initiation codon is difficult when several alternatives are inferred representing the start of coding sequence in a region. Most of PCGs initiate with the standard start codon ATG. Standard start codon ATA is used for *cox3 *in *C. virginica*, *nad4 *in *C. hongkongensis *and *nad4L *in *C. sikamea *and in *C. ariakensis*. Non-standard initiation codon GTG is used for *nad5 *in *C. ariakensis*, and ATT is used for *nad6 *in *C. hongkongensis *(Table 2). There are no obvious patterns in termination codon usage; the usage frequency of stop codon TAG is similar to that of TAA. Incomplete termination codons T and TA were also used. Termination codon TAA is used in 9 PCGs of *C. virginica*; if the incomplete termination codon is considered, the number of TAA is up to eleven. The PCGs with identical termination codon in the six genomes are *nad1 *and *nad4L*, which ended with TAA and incomplete stop codon T, respectively (Table 2).

**Table 2:** Comparison of gene length, initiation codon and termination codon in Crassostrea mt genomes

	*Cgi*	*Can*	*Csi*	*Cho*	*Car*	*Cvi*	*Cgi*	*Can*	*Csi*	*Cho*	*Car*	*Cvi*	*Cgi*	*Can*	*Csi*	*Cho*	*Cai*	*Cvi*
***cox1***	538	538	538	538	538	540	ATG	ATG	ATG	ATG	ATG	ATG	TAG	TAG	TAA	TAA	TAA	TAA
***cox2***	233	233	233	233	233	230	ATG	ATG	ATG	ATG	ATG	ATG	TAA	TAG	TAA	TAG	TAG	TAA
***cox3***	291	291	291	287	287	290	ATG	ATG	ATG	ATG	ATG	**ATA**	TAG	TAA	TAG	TAA	TAA	TA-
***nad1***	311	311	311	311	311	311	ATG	ATG	ATG	ATG	ATG	ATG	TAA	TAA	TAA	TAA	TAA	TAA
***nad2***	332	332	332	332	332	331	ATG	ATG	ATG	ATG	ATG	ATG	TAG	TAG	TAA	TAG	TAG	TAA
***nad3***	116	116	116	116	116	117	ATG	ATG	ATG	ATG	ATG	ATG	TAG	TAG	TAG	TAG	TAA	TAA
***nad4***	450	449	449	449	449	449	ATG	ATG	ATG	**ATA**	ATG	ATG	TAA	TAA	TAG	TAG	TAG	TAA
***nad4L***	94	94	94	93	94	93	ATG	ATG	**ATA**	ATG	**ATA**	ATG	T-	T-	T-	T-	T-	T-
***nad5***	556	556	557	556	556	555	ATG	ATG	ATG	ATG	**GTG**	ATG	TAG	TAG	TAG	TAA	TAG	TAA
***nad6***	158	158	158	158	159	153	ATG	ATG	ATG	ATT	ATG	ATG	TAG	TAG	TAG	TA-	TAA	TAA
***cob***	412	412	411	401	400	403	**CTA**	**CTA**	**CTG**	**ATA**	**TTA**	**TTA**	TAG	TAG	TAA	TAA	TAA	TAG
***atp6***	227	227	227	227	224	224	ATG	ATG	ATG	ATG	ATG	ATG	TAA	TAA	TAG	TAG	TAG	TAA
rrnL_1_*	601	602	602	605	606	748												
rrnL_2_*	713	713	712	712	712	721												
rrnS_1_*	1037	1038	1037	1074	1070	989												
rrnS_2_*	1205	1206	1195	1190	1183	0												

*rrnL_1 _and *rrnL_2 _*indicate the two parts of 5' half and 3'half *rrnL*, respectively; *rrnS_1 _*and *rrnS_2 _*indicate the duplication of *rrnS*.

The number of amino acids coded by each of the mt genomes is approximately equal. Excluding the stop codons, the *C. virginica *mtDNA encodes the least amino acids (3,696), while the *C. gigas *mtDNA encodes the most amino acids (3,718). Oysters with the lowest (60.1%) and highest (64.5%) A+T compositions of protein-coding region are *C. virginica *and *C. hongkongensis*, respectively. Similarly, oysters showing the lowest and highest A+T content of the first and the third positions are also *C. virginica *and *C. hongkongensis*: 55.0% and 58.3% for the first position, and 64.7% and 75.3% for the third position, respectively. However, the A+T content of the second position is approximately the same in the six genomes ranging from 60.0% in *C. hongkongensis *to 60.5% in *C. virginica*. It is obvious that the A+T content of the third codon position is higher than that of the first and the second positions. The genomic features of 19 bivalve sequences are presented in Table 1. The statistics of A+T content, start and stop codon, and amino acid number of PCGs in *C. iredalei *is already described in Wu et al's paper [54].

### Nonsynonymous and synonymous substitutions

**Figure 3:**
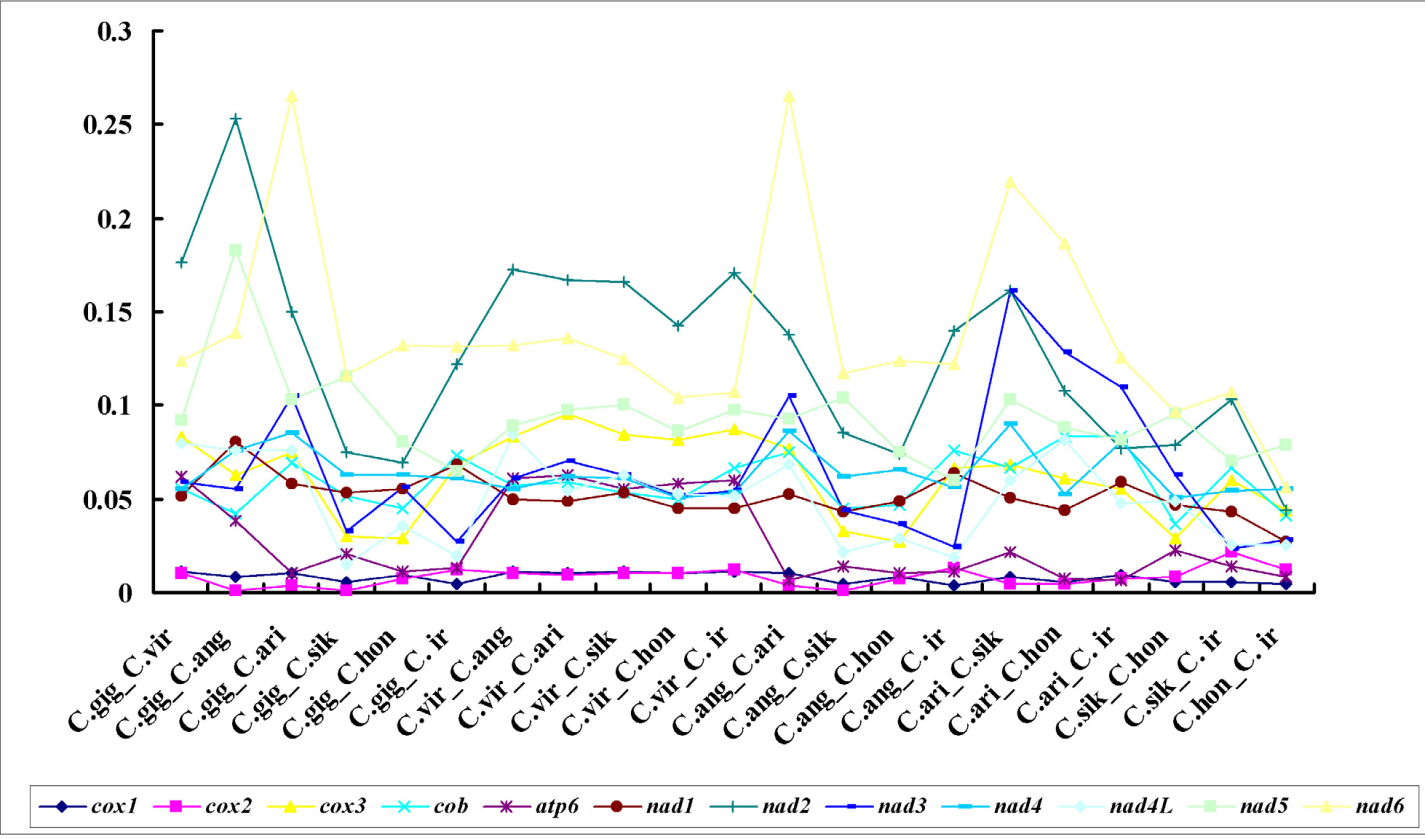
**The ratio of nonsynonymous and synonymous substitutions (Ka/Ks) estimated in all 12 protein genes of seven *Crassostrea *oysters**.

The estimation of nonsynonymous (Ka) and synonymous (Ks) substitution rates is of great significance in understanding evolutionary dynamics of protein-coding sequences across closely related species [55,56]. To detect the influence of selection pressure in *Crassostrea *species, the numbers of Ka and Ks were calculated and their ratios were plotted for all pairwise comparisons among the seven oysters (Figure 3 and additional file 2: Table S2). The ratio of Ka/Ks in all 12 protein-coding genes varied from 0.001 for *cox2 *in *C. angulata*_*C. sikamea *and *C. gigas*_*C. sikamea *to 0.2657 for *nad6 *in *C. angulata*_*C. ariakensis*, which supports the existence of different mutation constraints among genes. Most of the amino acid substitutions are localized in the NADH complex genes. It suggests a relaxation of purifying selection in the *nad6*, nad2, *nad3*, *nad5 *genes compared with the more conservative genes such as *cox1*, *cox2 *and *atp6*.

### Transfer and ribosomal RNA genes

The mt genomes of the six Asian *Crassostrea *oysters encode 25 tRNA genes. In addition to the 22 typical tRNAs, there are three duplications of *trnM*, *trnK *and *trnQ*. In comparison, however, there is no duplication of *trnK *and *trnQ *in *C. virginica*. The tRNA structure of *C. gigas *is referred to as the standard when the nucleotide variation in other four Asian oysters is examined (*C. iredalei *is not examined as it was not sequenced in this study). Nucleotide variations including transition/transversion and insertion/deletion mostly occur at DHU and TΨC loops (Additional file 3: Figure S1).

Duplication of tRNA is common in molluscs. A second *trnM *is also presented in the mussel *Mytilus edulis*, *M. galloprovincialis *and *M. trossulus *[45-47], and the clam *V. philippinarum*, *H. arctica *and *A. tuberculata *[48]. In *P. magellanicus*, there are up to ten copies of *trnM *[42]. In *M. trossulus *mtDNA, an additional copy of *trnQ *has also been reported [47]. The anticodon usage of oyster genomes is congruent to the corresponding tRNA of other molluscs with one exception. The anticodon of two *trnM*s in the oysters is CAU while that of two *trnM*s is CAU and UAU in *Mytilus*. The difference in anticodon corresponds to the third wobble position.

The boundaries of both the small and the large ribosomal genes were determined by BLAST with the revised annotation of *C. gigas *genome [23]. The *rrnL *gene is split into two segments, one segment of 5' end is distributed between *trnQ1 *and *trnN*, and the other segment of 3' end is located between *cox1 *and *cox3*. The split *rrnL *is first reported in *C. virginica*, but so far not reported in other metazoan mt genomes. The mt genome of *S. mordax *also has the split *rrnL*, however, it does not have the duplicated *rrnS*. The duplication of *rrnS *is only found in the six Asian *Crassostrea *oysters and the similarity between intraspecies and interspecies is high (95-100%) in the conserved 940 nucleotides at the center of *rrnS *sequences. The size of *rrnL *in the Asian *Crassostrea *oysters is nearly equal, and smaller than that in *C. virginica *(Table 1). The length of *rrnS *in *C. virginica *is 989 bp, while total length of *rrnS *in the Asian *Crassostrea *oysters varies from 2,232 bp (*C. sikamea*) to 2,264 bp (*C. hongkongensis*).

### Non-coding regions

As in most bivalves, the oyster mtDNAs contain a large number of unassigned nucleotides. There are more than 30 non-coding regions throughout the seven *Crassostrea *genomes. The unassigned nucleotides vary from 1,788 bp in *C. gigas *to 5,873 bp in *C. iredalei*. The proportion of the unassigned nucleotides in the whole genome varies from 9.81% in *C. gigas *to 26.17% in *C. iredalei*. The largest non-coding region in the Asian oysters is located between gene *trnG *and *trnV*, while it is between gene *trnP *and *trnG *in *C. virginica*. The sequence of each corresponding non-coding region from the six Asian *Crassostrea *oysters was listed separately and regions large than 30 nucleotides were aligned using ClustalX 1.83. There are 14 non-coding regions aligned (See the Supplement to the non-coding regions). The alignment shows that there are sequence conservation in the non-coding region, especially, higher sequence conservation in the regions between *trns2 *and *trnP*, *trnV *and *nad2*. The sequence similarity among species in the non-coding region is positively correlated with their relatedness.

### Phylogenetic analysis and divergence time estimation

**Figure 4:**
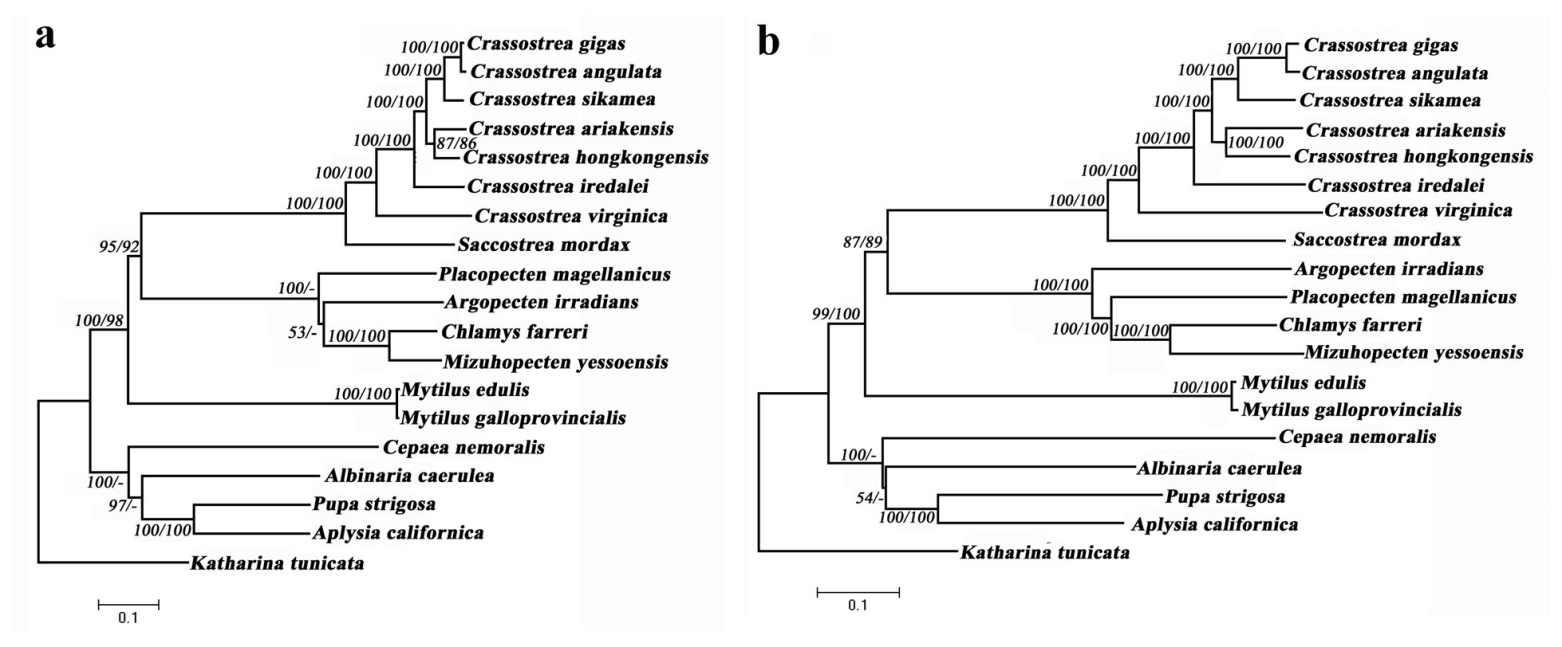
**Phylogenetic trees based on the concatenated amino acid (a) and nucleotide sequences (b) of 12 protein-coding genes (The branch length determined with NJ analysis)**. The chiton *Katharina tunicata *was used as outgroup. NJ (left number) and ML (right number) bootstrap values are given for each branch.

Phylogenetic analysis using all mt coding sequences provides clear evolutionary relationships among the seven *Crassostrea *and one *Saccostrea *oyster species (Figure 4). *C. gigas *is first clustered with *C. angulata *and then united with *C. sikamea*, meanwhile *C. hongkongensis *and *C. ariakensis *formed a clade. Finally, *C. iredalei *with other five *Crassostrea *oysters form a large Asian clade. Together with Asian *Crassostrea *oysters, the *C. virginica *formed the *Crassostrea *clade. *S. mordax *diverged early from *Crassostrea *oysters and was positioned at the base of the large oyster clade. All the phylogenetic relationships are supported with high values. The close relationships between *C. gigas *and *C. angulata*, and *C. hongkongensis *and *C. ariakensis *are clearly demonstrated on the phylogenetic trees (Figure 4), which has been the subject of debate for some time [14,12,22]. The complete mt genomes of *C. gigas *and *C. angulata *differed by 3%, providing strong support for their status as two independent species.

**Figure 5:**
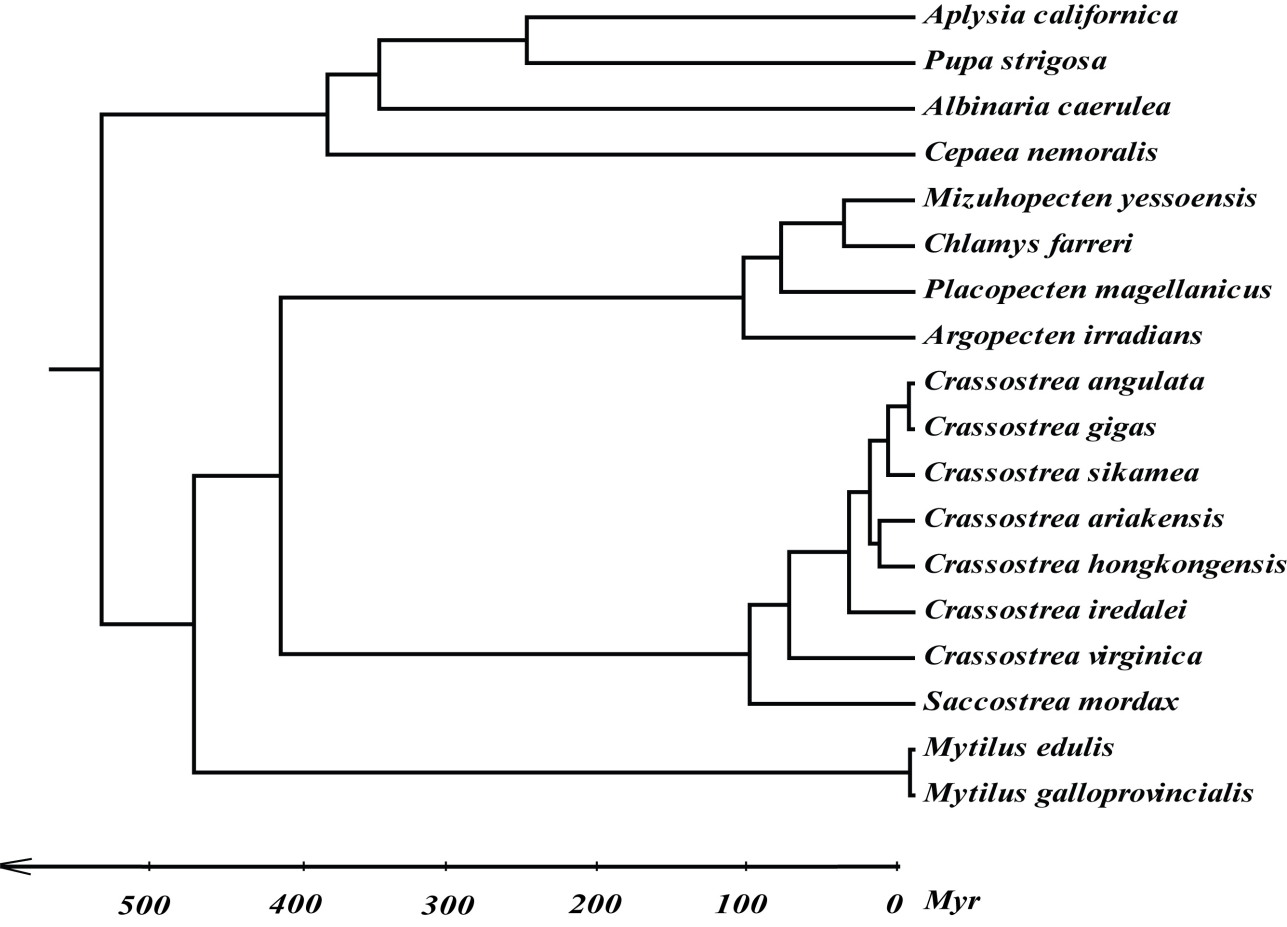
**A phylogenetic tree with divergence time (in million years) of 19 molluscan species rooted with the chiton *Katharina tunicata *as determined by Bayesian phylogenetic analysis**.

The first appearance of Gastropoda and Bivalvia in fossil record is approximately 542 Myr ago [40], providing the calibration constraint for divergence estimation. Divergence time estimates between species and/or clades are given in Table 3 and Figure 5. Our estimation shows that *C. virginica *and the Asian oysters diverged about 82.7 Myr ago. The six Asian *Crassostrea *oysters started their divergence about 42.8 Myr ago with the separation of *C. iredalei *from other species first. Other two large clades *C. hongkongensis *and *C. ariakensis*, and *C. sikamea*, *C. gigas *and *C. angulata *diverged 28.8 Myr ago. *C. hongkongensis *and *C. ariakensis *diverged 22.3 Myr ago. In the other clade, *C. sikamea *diverged from *C. gigas *and *C. angulata *16.5 Myr ago. The two most closely related species, *C. angulata *and *C. gigas*, diverged about 2.7 Myr ago.

**Table 3:** Optima (minima and maxima) in millions of years derived for each clade

Clade name	Mean (Myr)	SD (Myr)	Minima (Myr)	Maxima (Myr)
*Aplysia californica *: *Pupa strigosa*	257.434	18.841	220.719	295.515
*Albinaria caerulea*: (*Aplysia californica *: *Pupa strigosa*)	355.753	18.063	320.518	391.260
*Cepaea nemoralis *: (*Albinaria caerulea *: (*Aplysia californica *: *Pupa strigosa*))	390.342	18.392	354.283	426.306
*Mytilus edulis *: *Mytilus galloprovincialis*	2.474	0.553	1.556	3.717
*Chlamys farreri *: *Mizuhopecten yessoensis*	46.142	5.483	36.418	57.765
*Placopecten magellanicus *: (*Chlamys farreri *: *Mizuhopecten yessoensis*)	88.175	9.537	70.950	108.724
*Argopecten irradians *: (*Placopecten magellanicus *: (*Chlamys farreri *: *Mizuhopecten yessoensis*))	113.296	11.374	92.757	137.153
				
*Crassostrea angulata *: *C. gigas*	2.723	0.397	2.025	3.577
*C. sikamea *: (*C. angulata *: *C. gigas*)	16.469	2.040	12.770	20.885
*C. hongkongensis *: *C. ariakensis*	22.316	2.786	17.333	28.334
(*C. hongkongensis *: *C. ariakensis*): (*C. sikamea *: (*C. angulata *: *C. gigas*))	28.781	3.448	22.615	36.222
*C. iredalei *: ((*C. hongkongensis *: *Cr. ariakensis*): (*C. sikamea *: (*C. angulata *: *C. gigas*)))	42.770	5.051	33.622	53.649
*C. virginica *: (*C. iredalei *: ((*C. hongkongensis *: *C. ariakensis*): (*C. sikamea *: (*C. angulata *: *C. gigas*))))	82.651	9.015	66.209	101.967
*Saccostrea mordax *(*C. virginica *: (*C. iredalei *: ((*C. hongkongensis *: *C. ariakensis*): (*C. sikamea *: (*C. angulata *: *C. gigas*)))))	109.049	11.125	88.800	132.464
Ostreoida: Pectinoida	421.475	18.890	384.824	4.58.57
Mytiloida: (Ostreoida: Pectinoida)	479.361	16.121	448.075	510.635
Gastropoda: Bivalvia	540.866	11.359	522.899	560.657

These estimates indicate that, relative to the divergence with *C. virginica *about 83 Myr ago, the speciation of the six Asian *Crassostrea *species is rather recent or 3-43 Myr ago. Still, it is difficult to reconcile molecular divergence with genome rearrangement data observed here. Assuming the divergence time estimates are correct, it is intriguing that the mt genomes of *C. virginica *and the Asian species diverged with nine structure rearrangements (6 transpositions and 3 duplications) in about 83 Myr, while there was not a single rearrangement event among the six Asian *Crassostrea *species in the past 43 Myr. During the same time frame, numerous rearrangement events occurred among scallop species (Figure 2). Thus, the complete conservation of gene order among the Asian *Crassostrea *oysters in the past 43 Myr is highly unusual, given the amazing rate of mt genome rearrangements in marine bivalves in general and within the genus *Crassostrea *itself. While this finding provides strong evidence for recent speciation of the six Asian *Crassostrea *species, it also indicates that mt genome rearrangement may not be strictly a function of time, but constrained by other factors. Detailed mechanisms of mt genome rearrangement are unknown. We suspect that coding on both strands may be one of the factors inhibiting mt genome rearrangement. This hypothesis is supported by the fact that the only two bivalve molluscs (*L. ornata *and *I. japanensis*) with dual-strand coding have relatively fewer rearrangements of gene order compared with other molluscan classes (Figure 2). All marine bivalves use one strand for coding and show tremendous rearrangements. The extensive rearrangement of bivalve mt genomes may be a consequence of coding on one strand. Furthermore, the fact that massive rearrangements in marine bivalves have not disrupted single-strand coding or changed the direction of transcription suggests that the rearrangements are not caused by inversion or reverse transposition. Thus, transposition or "tandem repeat-random loss"[49] may be important for mt genome rearrangement [29].

Dual-strand coding may not be the only factor limiting mt genome rearrangement, as it cannot explain the absence of rearrangements in the six Asian oysters over 43 Myr. We speculate that some other unknown features in mt genomes of the six Asia species may also limit genome rearrangements. The most noticeable structure change is the duplication of *rrnS *in the six Asian species, though it is difficult to determine if it has any effects on genome rearrangement at current stage. Further studies on the mt genomes of other oysters and marine bivalves may shed light on the origin and evolution of genome rearrangements. Gene order data have been shown to be valuable in phylogenetic analysis [3-6]. While our analysis demonstrates the power of rearrangement data, it also argues for a better understanding of mt genome rearrangement before using them to infer divergence time.

## Conclusions

Asia Pacific has long been suspected to be the center of oyster speciation. Our analysis of complete mt genomes provides strong evidence that *Crassostrea *oysters have diversified in Asia and the divergence was rather recent or within the last 3-43 Myr. The complete conservation of gene order in the six Asian *Crassostrea *species over a period of 43 Myr is highly unusual given the remarkable rate of rearrangements in their sister species and other bivalves during the same time frame. It provides strong evidence, in addition to sequence data, for the recent speciation of *Crassostrea *oysters in Asia. It also indicates that changes in mt gene order may not be strictly a function of time but subject to other constraints that are presently not well understood.

## Methods

### Sample collection and DNA extraction

Specimens for the five Asian *Crassostrea *species were mostly collected from coastal waters of China, except that *C. gigas *was collected from a cultured population in Oregon, USA. *C. gigas *is a native species of Asia and was introduced to the West Coast of USA for aquaculture production. The oyster ID, geographic origin and collection date of each specimen are provided in Table 4. All oysters were identified according to their morphological characteristics first and confirmed with species-specific molecular markers [21]. Total genomic DNA was extracted from ethanol-fixed tissue with the CTAB method and dissolved in TE (10 mM Tris-HCl 1 mM EDTA pH 8.0) buffer before being stored at -20°C.

**Table 4:** Sample information for the five *Crassostrea *species studied

Species	Oyster ID	Origin	Date
*C. gigas*	ORCg-4	Cultured, Oregon, USA	06/2005
*C. angulata*	Cangtaiwh-9	Wild, Taiwan, China	06/2006
*C. sikamea*	NT-521	Wild, Nantong, Jiangsu, China	04/2006
C. hongkongensis*	Hainan big#1	Cultured, Lingao, Hainan, China	08/2005
*C. ariakensis*	YKy101	Wild, Yingkou, Liaoning, China	08/2006

* This data has been published in Ren et al., 2009 [25]

### PCR amplification and DNA sequencing

Four pairs of primers were designed to amplify the complete mt genomes of *C. gigas *and *C. angulata *according to the sequence of *C. gigas *in GenBank (Additional file 3: Table S3). Sequencing primers were designed at intervals of about 500 bp. All PCR products were directly sequenced by primer-walking. Partial sequences of *C. sikamea *and *C. ariakensis *were obtained with the combined primers of the amplifying and sequencing primers designed for *C. gigas*. The remaining gaps were amplified with the species-specific primers designed according to the obtained sequences. As for *C. hongkongensis*, two short fragments of *cox1 *and *cox2 *were first amplified with the universal primer sets of LCO1490+ HCO2198 [53] and cox2F+cox2R [26,27]. In addition, partial sequence of *nad5 *was amplified with the primers of *C. gigas*. Then, the whole mt genome was amplified based on three pairs of primers (Additional file 4: Table S3).

PCR reactions were performed with a Mastercycler gradient machine (Eppendorf). The cycling was set up with an initial denature step at 94°C for 2 min, followed by 35 cycles comprising denaturing at 94°C for 20 sec, annealing at 52-58°C for 1 min and elongation at 68°C or 72°C for 6 or 10 min depending on the expected length of the PCR products. The process was completed with a final elongation at 72°C for 10 min. The reaction volume amounted 25 μl containing 18.8 μl sterile deionized water, 2.5 μl 10×LA PCR buffer (Mg2+ plus, Takara), 1 μl dNTP mix (10 mM each), 1 μl each primer (5 μM), 0.2 μl LA Taq DNA polymerase (5 U/μl, Takara) and 0.5 μl DNA template (50 ng/μl). A negative control (no template) was included during each PCR run. PCR products were directly purified with MultiScreen-PCR96 Filter Plate (Millipore) and sequenced with ABI 3730x1 DNA Analyzer (Applied Biosystems).

### Sequence analysis and gene annotation

Raw sequencing reads were first processed using Phred with the quality score 20 and assembled in Phrap with default parameters [28,29]. Then, all assemblies and sequence quality were verified manually in Consed to remove misassemblies [30]. The accurate boundary of each gene was determined according to the annotated *C. gigas *mt genome [23] with minor revisions. The tRNA genes were identified by tRNAscan-SE 1.21 [31] employing the cove only search mode and the invertebrate mt genetic code. The ratio of nonsynonymous and synonymous substitutions rates (Ka/Ks) was estimated with Ka_Ks calculator in all 12 protein genes of seven *Crassostrea *oysters.

### Phylogeny analysis and divergence time estimation

Nineteen molluscan mt genomes including four obtained in this study were used for phylogenetic analysis (Table 5). The black chiton *Katharina tunicata *(Polyplacophora) was rooted as the outgroup. The amino acid sequence from each of 12 protein-coding genes (excluding *atp8*) was aligned separately using ClustalX 1.83 [33], and then trimmed to the same length and concatenated for further analysis. The nucleotide sequence was substituted from the concatenated amino acid alignment. The final nucleotide sequence consisted of 9,537 sites. Two phylogenetic reconstruction approaches were performed including Maximum Likelihood (ML) with PHYML 3.0 [34] and Neighbor-Joining (NJ) with MEGA 4.0 [35]. The assessments of node reliability in both the ML and NJ analyses were done by using 1,000 bootstrap replicates.

**Table 5:** List of taxa used in the phylogenetic analysis

Taxon	Classification	GenBank Accession Number
**Polyplacophora**		
*Katharina tunicata*	Neoloricata; Chitonida; Acanthochitonina; Mopaliidae	NC_001636
**Gastropoda**		
*Aplysia californica*	Orthogastropoda; Apogastropoda; Heterobranchia; Euthyneura; Opisthobranchia; Anaspidea; Aplysioidea; Aplysiidae;	NC_005827
*Pupa strigosa*	Orthogastropoda;Apogastropoda; Heterobranchia; Euthyneura; Opisthobranchia; Architectibranchia; Acteonoidea; Acteonidae	NC_002176
*Albinaria caerulea*	Pulmonata; Stylommatophora; Sigmurethra; Clausilioidea; Clausiliidae	NC_001761
*Cepaea nemoralis*	Pulmonata; Stylommatophora; Sigmurethra; Helicoidea; Helicidae	NC_001816
**Bivalvia**		
*Mytilus galloprovincialis*	Bivalvia; Pteriomorphia; Mytiloida;Mytiloidea; Mytilidae	NC_006886
*Mytilus edulis*	Bivalvia; Pteriomorphia; Mytiloida; Mytiloidea; Mytilidae	NC_006161
*Argopecten irradians*	Bivalvia; Pteriomorphia; Pectinoida;Pectinoidea; Pectinidae	
*Placopecten magellanicus*	Bivalvia; Pteriomorphia; Pectinoida;Pectinoidea; Pectinidae	NC_007234
*Mizuhopecten yessoensis*	Bivalvia; Pteriomorphia; Pectinoida;Pectinoidea; Pectinidae	NC_009081
*Chlamys farreri*	Bivalvia; Pteriomorphia; Pectinoida;Pectinoidea; Pectinidae	
*Crassostrea virginica*	Bivalvia; Pteriomorphia; Ostreoida;Ostreoidea; Ostreidae	NC_007175
*Crassostrea gigas*	Bivalvia; Pteriomorphia; Ostreoida;Ostreoidea; Ostreidae	EU672831
*Crassostrea angulata*	Bivalvia; Pteriomorphia; Ostreoida;Ostreoidea; Ostreidae	NC_012648
*Crassostrea sikamea*	Bivalvia; Pteriomorphia; Ostreoida;Ostreoidea; Ostreidae	NC_012649
*Crassostrea hongkongensis*	Bivalvia; Pteriomorphia; Ostreoida;Ostreoidea; Ostreidae	NC_011518
*Crassostrea ariakensis*	Bivalvia; Pteriomorphia; Ostreoida;Ostreoidea; Ostreidae	NC_012650
*Crassostrea iredalei*	Bivalvia; Pteriomorphia; Ostreoida;Ostreoidea; Ostreidae	NC_013997
*Saccostrea mordax*	Bivalvia; Pteriomorphia; Ostreoida;Ostreoidea; Ostreidae	NC_013998

Molecular estimates of divergence time for multiple gene data were performed using the relaxed Bayesian molecular clock approach as implemented in MULTIDISTRIBUTE package [36]. Key features of this program are that posterior distribution of molecular time estimates and rates of molecular evolution are approximated while simultaneously taking account of uncertainty in branch length estimates from each gene [37]. The F84+gamma evolution model, incorporating different rates of transition/transversion, variable nucleotide frequencies, and nucleotide variation across sites, was used to estimate the maximum likelihood parameter in PAML version 3.15 [38]. The Multidivtime program used the output from Estbranches analyses to estimate node divergence times for the ingroup, given time constraints, various parameters, and estimated priors. The median of all the tip-to-root branch lengths was calculated using ape and LAGOPUS package in R project [39]. Gamma priors were chosen as the following procedure outlined in the Multidivtime manual: expected time between the tip and the ingroup root (rttime) = 542 Myr (million years) ago, with standard deviation (SD) = 30 Myr ago; rate of the root node (rtrate) and its SD = 0.46 substitution per site per 100 Myr determined as the median of all the tip-to-root branch lengths divided by rttime; and rate of change between ancestral and descendant nodes (brownmean) = 0.18. *Katharina tunicata *was considered to be outgroup to Bivalvia and Gastropoda as required by the program. All divergence time were calculated assuming the topology of the consensus tree, which was derived from previous ML/Bayesian analyses based on protein sequences. The first appearance of skeletons in the fossil record, indicating the maximum for the origin of Gastropoda + Bivalvia is approximately 542 Myr ago, provides the calibration constraint for divergence estimation [40]. The parameters for the Markov Chain Monte Carlo (MCMC) simulation were set as follows: number of samples = 10,000, sample frequency = 200 and burn-in period = 2,000. Finally, Multidivtime analyses considering variance-covariance matrices from each gene partition were run twice ensure convergence, each one starting with a different random initial seed number.

## Authors' contributions

JR did PCR, sequencing and initial analysis; FJ did some data analysis; XG and XL provided the samples; BL, XG and XL conceived the study; JR, BL and XG wrote and revised the manuscript. All authors read and approved the final manuscript.

## Supplementary Material

Additional file 1 **Table S1: Organization of the mitochondrial genome of six oysters**. Click here for file

Additional file 2 **Table S2: The ratio of nonsynonymous and synonymous substitutions (Ka/Ks) estimated with Ka_Ks calculator in all 12 protein genes of seven Crassostrea oysters**. Click here for file

Additional file 3 **Figure S1: Comparison of the potential secondary structures of the 25 inferred tRNAs among five *Crassostrea *oyster mtDNAs**. Click here for file

Additional file 4 **Table S3: Major primers used in amplifying the mitochondrial genomes**. Click here for file
