# Supplementary material for: Unusual conservation of mitochondrial gene order in Crassostrea oysters: evidence for recent speciation in Asia
Source: BMC Evol Biol. 2010 Dec 28;10:394. doi: 10.1186/1471-2148-10-394 (PMC3040558; doi:10.1186/1471-2148-10-394)
Supplement: Additional file 1 — Table S1: Organization of the mitochondrial genome of six oysters. [file 1471-2148-10-394-S1.DOC]

*Table S1-1* *Organization of the mitochondrial genome of* C. gigas

| Gene | Position | Size  Nucleotide Amino acid | | Codon  Start Stop | | Intergenic  Nucleotides* |
| --- | --- | --- | --- | --- | --- | --- |
| ***cox1*** | 1-1617 | 1617 | 538 | ATG | TAG | 4 |
| ***rrnL*** 3’ half | 1767-2479 | 713 |  |  |  | 149 |
| ***cox3*** | 2586-3461 | 876 | 291 | ATG | TAG | 105 |
| ***trnI*** | 3462-3529 | 68 |  |  |  | 0 |
| ***trnT*** | 3530-3597 | 68 |  |  |  | 0 |
| ***trnE*** | 3618-3686 | 69 |  |  |  | 20 |
| ***cob*** | 3689-4927 | 1239 | 412 | CTA | TAG | 2 |
| ***trnD*** | 5043-5111 | 69 |  |  |  | 115 |
| ***cox2*** | 5113-5814 | 702 | 233 | ATG | TAA | 1 |
| ***trnM_1_*(*^ATG^*)** | 5835-5900 | 66 |  |  |  | 20 |
| ***trnS_1_*(*^AGN^*)** | 5907-5976 | 70 |  |  |  | 6 |
| ***trnL_2_*(*^UUR^*)** | 5993-6059 | 67 |  |  |  | 16 |
| ***trnM_2_*(*^ATG^*)** | 6128-6192 | 65 |  |  |  | 68 |
| ***trnS_2_*(*^UCN^*)** | 6200-6267 | 68 |  |  |  | 7 |
| ***trnP*** | 6444-6512 | 69 |  |  |  | 176 |
| ***rrnS_1_*** | 6513-7549 | 1037 |  |  |  | 0 |
| ***trnK_1_*(*^AAA^*)** | 7550-7618 | 69 |  |  |  | 0 |
| ***trnC*** | 7643-7710 | 68 |  |  |  | 24 |
| ***trnQ_1_*(*^CAA^*)** | 7761-7829 | 69 |  |  |  | 50 |
| ***rrnL*** 5’ half | 7830-8430 | 601 |  |  |  | 0 |
| ***trnN*** | 8481-8546 | 66 |  |  |  | 50 |
| ***rrnS_2_*** | 8547-9751 | 1205 |  |  |  | 0 |
| ***trnY*** | 9752-9816 | 65 |  |  |  | 0 |
| ***atp6*** | 9822-10505 | 684 | 227 | ATG | TAA | 5 |
| ***trnG*** | 10541-10610 | 70 |  |  |  | 35 |
| ***trnV*** | 11256-11322 | 67 |  |  |  | 645 |
| ***nad2*** | 11364-12362 | 999 | 332 | ATG | TAG | 41 |
| ***trnR*** | 12402-12468 | 67 |  |  |  | 39 |
| ***trnH*** | 12528-12592 | 65 |  |  |  | 59 |
| ***nad4*** | 12595-13947 | 1353 | 500 | ATG | TAA | 2 |
| ***trnK_2_*(*^AAA^*)** | 13956-14023 | 68 |  |  |  | 8 |
| ***nad5*** | 14025-15695 | 1671 | 556 | ATG | TAG | 1 |
| ***nad6*** | 15708-16184 | 477 | 158 | ATG | TAG | 12 |
| ***trnQ_2_*(*^CAA^*)** | 16219-16288 | 70 |  |  |  | 34 |
| ***nad3*** | 16293-16643 | 351 | 116 | ATG | TAG | 4 |
| ***trnL_1_*(*^CUN^*)** | 16678-16743 | 66 |  |  |  | 34 |
| ***trnF*** | 16779-16846 | 68 |  |  |  | 35 |
| ***trnA*** | 16862-16928 | 67 |  |  |  | 15 |
| ***nad1*** | 16933-17868 | 936 | 311 | ATG | TAA | 4 |
| ***nad4L*** | 17870-18152 | 283 | 94 | ATG | T- | 1 |
| ***trnW*** | 18153-18221 | 69 |  |  |  | 0 |

Table S1-2 Organization of the mitochondrial genome of *C. angulata*

| Gene | Position | Size  Nucleotide Amino acid | | Codon  Start Stop | | Intergenic  Nucleotide* |
| --- | --- | --- | --- | --- | --- | --- |
| ***cox1*** | 1-1617 | 1617 | 538 | ATG | TAG | 4 |
| ***rrnL***3’ half | 1766-2478 | 713 |  |  |  | 148 |
| ***cox3*** | 2586-3461 | 876 | 291 | ATG | TAA | 107 |
| ***trnI*** | 3462-3529 | 68 |  |  |  | 0 |
| ***trnT*** | 3530-3597 | 68 |  |  |  | 0 |
| ***trnE*** | 3618-3686 | 69 |  |  |  | 20 |
| ***cob*** | 3689-4927 | 1239 | 412 | CTA | TAG | 2 |
| ***trnD*** | 5043-5111 | 69 |  |  |  | 115 |
| ***cox2*** | 5113-5814 | 702 | 233 | ATG | TAG | 1 |
| ***trnM_1_*(*^ATG^*)** | 5835-5900 | 66 |  |  |  | 20 |
| ***trnS_1_*(*^AGN^*)** | 5907-5976 | 70 |  |  |  | 6 |
| ***trnL_2_*(*^UUR^*)** | 5993-6059 | 67 |  |  |  | 16 |
| ***trnM_2_*(*^ATG^*)** | 6127-6191 | 65 |  |  |  | 67 |
| ***trnS_2_*(*^UCN^*)** | 6199-6266 | 68 |  |  |  | 7 |
| ***trnP*** | 6443-6511 | 69 |  |  |  | 176 |
| ***rrnS_1_*** | 6512-7549 | 1038 |  |  |  | 0 |
| ***trnK_1_*(*^AAA^*)** | 7550-7618 | 69 |  |  |  | 0 |
| ***trnC*** | 7643-7710 | 68 |  |  |  | 24 |
| ***trnQ_1_*(*^CAA^*)** | 7761-7829 | 69 |  |  |  | 50 |
| ***rrnL*** 5’half | 7830-8431 | 602 |  |  |  | 0 |
| ***trnN*** | 8482-8547 | 66 |  |  |  | 50 |
| ***rrnS_2_*** | 8548-9753 | 1206 |  |  |  | 0 |
| ***trnY*** | 9754-9818 | 65 |  |  |  | 0 |
| ***atp6*** | 9824-10507 | 684 | 227 | ATG | TAA | 5 |
| ***trnG*** | 10543-10612 | 70 |  |  |  | 35 |
| ***trnV*** | 11256-11322 | 67 |  |  |  | 643 |
| ***nad2*** | 11364-12362 | 999 | 332 | ATG | TAG | 41 |
| ***trnR*** | 12402-12468 | 67 |  |  |  | 39 |
| ***trnH*** | 12528-12592 | 65 |  |  |  | 59 |
| ***nad4*** | 12597-13946 | 1350 | 499 | ATG | TAA | 4 |
| ***trnK_2_*(*^AAA^*)** | 13955-14022 | 68 |  |  |  | 8 |
| ***nad5*** | 14024-15694 | 1671 | 556 | ATG | TAG | 1 |
| ***nad6*** | 15707-16183 | 477 | 158 | ATG | TAG | 12 |
| ***trnQ_2_*(*^CAA^*)** | 16219-16288 | 70 |  |  |  | 35 |
| ***nad3*** | 16293-16643 | 351 | 116 | ATG | TAG | 4 |
| ***trnL_1_*(*^CUN^*)** | 16678-16743 | 66 |  |  |  | 34 |
| ***trnF*** | 16780-16845 | 66 |  |  |  | 36 |
| ***trnA*** | 16862-16928 | 67 |  |  |  | 16 |
| ***nad1*** | 16933-17868 | 936 | 311 | ATG | TAA | 4 |
| ***nad4L*** | 17870-18152 | 283 | 94 | ATG | T- | 1 |
| ***trnW*** | 18153-18221 | 69 |  |  |  | 0 |

Table S1-3 Organization of the mitochondrial genome of *C. sikamea*

| Gene | Position | Size  Nucleotide Amino acid | | Codon  Start Stop | | Intergenic  Nucleotides* |
| --- | --- | --- | --- | --- | --- | --- |
| ***cox1*** | 1-1617 | 1617 | 538 | ATG | TAA | 4 |
| ***rrnL*** 3’half | 1763-2474 | 712 |  |  |  | 145 |
| ***cox3*** | 2583-3458 | 876 | 291 | ATG | TAG | 108 |
| ***trnI*** | 3459-3525 | 67 |  |  |  | 0 |
| ***trnT*** | 3526-3593 | 68 |  |  |  | 0 |
| ***trnE*** | 3614-3682 | 69 |  |  |  | 20 |
| ***cob*** | 3685-4920 | 1236 | 411 | ATG | TAA | 2 |
| ***trnD*** | 5049-5117 | 69 |  |  |  | 128 |
| ***cox2*** | 5119-5820 | 702 | 233 | ATG | TAA | 1 |
| ***trnM_1_*(*^ATG^*)** | 5841-5906 | 66 |  |  |  | 20 |
| ***trnS_1_*(*^AGN^*)** | 5916-5985 | 70 |  |  |  | 9 |
| ***trnL_2_*(*^UUR^*)** | 6001-6067 | 67 |  |  |  | 15 |
| ***trnM_2_*(*^ATG^*)** | 6133-6197 | 65 |  |  |  | 65 |
| ***trnS_2_*(*^UCN^*)** | 6205-6272 | 68 |  |  |  | 7 |
| ***trnP*** | 6450-6518 | 69 |  |  |  | 177 |
| ***rrnS_1_*** | 6519-7555 | 1037 |  |  |  | 0 |
| ***trnK_1_*(*^AAA^*)** | 7556-7624 | 69 |  |  |  | 0 |
| ***trnC*** | 7648-7715 | 68 |  |  |  | 23 |
| ***trnQ_1_*(*^CAA^*)** | 7762-7831 | 70 |  |  |  | 46 |
| ***rrnL*** 5’half | 7833-8434 | 602 |  |  |  | 1 |
| ***trnN*** | 8493-8558 | 66 |  |  |  | 58 |
| ***rrnS_2_*** | 8559-9753 | 1195 |  |  |  | 0 |
| ***trnY*** | 9754-9818 | 65 |  |  |  | 0 |
| ***atp6*** | 9824-10507 | 684 | 227 | ATG | TAG | 5 |
| ***trnG*** | 10545-10614 | 70 |  |  |  | 37 |
| ***trnV*** | 11270-11336 | 67 |  |  |  | 655 |
| ***nad2*** | 11380-12378 | 999 | 332 | ATG | TAA | 43 |
| ***trnR*** | 12417-12483 | 67 |  |  |  | 38 |
| ***trnH*** | 12544-12608 | 65 |  |  |  | 60 |
| ***nad4*** | 12611-13960 | 1350 | 449 | ATG | TAG | 2 |
| ***trnK_2_*(*^AAA^*)** | 13969-14037 | 69 |  |  |  | 8 |
| ***nad5*** | 14039-15712 | 1674 | 557 | ATG | TAG | 1 |
| ***nad6*** | 15726-16202 | 477 | 158 | ATG | TAG | 13 |
| ***trnQ_2_*(*^CAA^*)** | 16238-16307 | 70 |  |  |  | 35 |
| ***nad3*** | 16312-16662 | 351 | 116 | ATG | TAG | 4 |
| ***trnL_1_*(*^CUN^*)** | 16697-16762 | 66 |  |  |  | 34 |
| ***trnF*** | 16794-16861 | 68 |  |  |  | 31 |
| ***trnA*** | 16879-16945 | 67 |  |  |  | 17 |
| ***nad1*** | 16950-17885 | 936 | 311 | ATG | TAA | 4 |
| ***nad4L*** | 17888-18170 | 283 | 94 | ATA | T- | 2 |
| ***trnW*** | 18171-18239 | 69 |  |  |  | 0 |

Table S1-4 Organization of the mitochondrial genome of *C. hongkongensis*

| Gene | Position | Size  Nucleotide Amino acids | | Codon  Start Stop | | Intergenic  Nucleotides* |
| --- | --- | --- | --- | --- | --- | --- |
| ***cox1*** | 1-1617 | 1617 | 538 | ATA | TAA | 1 |
| ***rrnL*** 3’half | 1761-2472 | 712 |  |  |  | 143 |
| ***cox3*** | 2575-3438 | 864 | 287 | ATA | TAA | 231 |
| ***trnI*** | 3439-3505 | 67 |  |  |  | 0 |
| ***trnT*** | 3506-3573 | 68 |  |  |  | 0 |
| ***trnE*** | 3595-3662 | 68 |  |  |  | 21 |
| ***cob*** | 3670-4875 | 1206 | 401 | ATA | TAA | 130 |
| ***trnD*** | 4984-5052 | 69 |  |  |  | 108 |
| ***cox2*** | 5054-5755 | 702 | 233 | ATG | TAG | 1 |
| ***trnM_1_*(*^ATG^*)** | 5777-5842 | 66 |  |  |  | 21 |
| ***trnS_1_*(*^AGN^*)** | 5846-5915 | 70 |  |  |  | 3 |
| ***trnL_2_*(*^UUR^*)** | 5931-5997 | 67 |  |  |  | 15 |
| ***trnM_2_*(*^ATG^*)** | 6065-6129 | 65 |  |  |  | 67 |
| ***trnS_2_*(*^UCN^*)** | 6137-6204 | 68 |  |  |  | 7 |
| ***trnP*** | 6383-6451 | 69 |  |  |  | 178 |
| ***rrnS_1_*** | 6452-7525 | 1074 |  |  |  | 0 |
| ***trnK_1_*** | 7526-7594 | 69 |  |  |  | 0 |
| ***trnC*** | 7624-7691 | 68 |  |  |  | 29 |
| ***trnQ_1_*(*^CAA^*)** | 7709-7777 | 69 |  |  |  | 17 |
| ***rrnL*** 5’half | 7780-8384 | 605 |  |  |  | 1 |
| ***trnN*** | 8443-8508 | 66 |  |  |  | 58 |
| ***rrnS_2_*** | 8509-9698 | 1190 |  |  |  | 0 |
| ***trnY*** | 9699-9764 | 66 |  |  |  | 0 |
| ***atp6*** | 9770-10453 | 684 | 227 | ATG | TAG | 5 |
| ***trnG*** | 10966-11035 | 70 |  |  |  | 512 |
| ***trnV*** | 11644-11716 | 73 |  |  |  | 608 |
| ***nad2*** | 11759-12757 | 999 | 332 | ATG | TAG | 42 |
| ***trnR*** | 12792-12858 | 67 |  |  |  | 34 |
| ***trnH*** | 12919-12983 | 65 |  |  |  | 60 |
| ***nad4*** | 12986-14335 | 1350 | 449 | ATA | TAG | 2 |
| ***trnK_2_*(*^AAA^*)** | 14343-14417 | 75 |  |  |  | 7 |
| ***nad5*** | 14419-16089 | 1671 | 556 | ATG | TAA | 1 |
| ***nad6*** | 16101-16576 | 476 | 158 | ATT | TA- | 11 |
| ***trnQ_2_*(*^CAA^*)** | 16610-16678 | 69 |  |  |  | 33 |
| ***nad3*** | 16684-17034 | 351 | 116 | ATG | TAG | 5 |
| ***trnL_1_*(*^CUN^*)** | 17070-17135 | 66 |  |  |  | 35 |
| ***trnF*** | 17171-17238 | 68 |  |  |  | 35 |
| ***trnA*** | 17259-17325 | 67 |  |  |  | 20 |
| ***nad1*** | 17331-18266 | 936 | 311 | ATG | TAA | 5 |
| ***nad4L*** | 18270-18549 | 280 | 93 | ATG | T- | 3 |
| ***trnW*** | 18550-18618 | 69 | 22 |  |  | 0 |

Table S1-5 Organization of the mitochondrial genome of *C. ariakensis*

| Gene | Position | Size  Nucleotide Amino acids | | Codon  Start Stop | | Intergenic  Nucleotides* |
| --- | --- | --- | --- | --- | --- | --- |
| ***cox1*** | 1-1617 | 1617 | 538 | ATG | TAA | 1 |
| ***rrnL*** 3’half | 1766-2477 | 712 |  |  |  | 148 |
| ***cox3*** | 2583-3446 | 864 | 287 | ATG | TAA | 105 |
| ***trnI*** | 3450-3516 | 67 |  |  |  | 3 |
| ***trnT*** | 3517-3584 | 68 |  |  |  | 0 |
| ***trnE*** | 3606-3674 | 69 |  |  |  | 21 |
| ***cob*** | 3685-4887 | 1203 | 400 | TTA | TAA | 10 |
| ***trnD*** | 4990-5058 | 69 |  |  |  | 102 |
| ***cox2*** | 5060-5761 | 702 | 233 | ATG | TAG | 1 |
| ***trnM_1_*(*^ATG^*)** | 5783-5848 | 66 |  |  |  | 21 |
| ***trnS_1_*(*^AGN^*)** | 5850-5919 | 70 |  |  |  | 1 |
| ***trnL_2_*(*^UUR^*)** | 5935-6001 | 67 |  |  |  | 15 |
| ***trnM_2_*(*^ATG^*)** | 6072-6137 | 66 |  |  |  | 70 |
| ***trnS_2_*(*^UCN^*)** | 6144-6211 | 68 |  |  |  | 6 |
| ***trnP*** | 6390-6458 | 69 |  |  |  | 178 |
| ***rrnS_1_*** | 6459-7528 | 1070 |  |  |  | 0 |
| ***trnK_1_*(*^AAA^*)** | 7529-7598 | 70 |  |  |  | 0 |
| ***trnC*** | 7621-7688 | 68 |  |  |  | 22 |
| ***trnQ_1_*(*^CAA^*)** | 7725-7793 | 69 |  |  |  | 36 |
| ***rrnL*** 5’half | 7796-8401 | 606 |  |  |  | 2 |
| ***trnN*** | 8453-8518 | 66 |  |  |  | 51 |
| ***rrnS_2_*** | 8519-9701 | 1183 |  |  |  | 0 |
| ***trnY*** | 9702-9767 | 66 |  |  |  | 0 |
| ***atp6*** | 9773-10447 | 675 | 224 | ATG | TAG | 5 |
| ***trnG*** | 10633-10701 | 69 |  |  |  | 185 |
| ***trnV*** | 11418-11487 | 70 |  |  |  | 716 |
| ***nad2*** | 11534-12532 | 999 | 332 | ATG | TAG | 46 |
| ***trnR*** | 12567-12633 | 67 |  |  |  | 34 |
| ***trnH*** | 12717-12781 | 65 |  |  |  | 83 |
| ***nad4*** | 12784-14133 | 1350 | 449 | ATG | TAG | 2 |
| ***trnK_2_*(*^AAA^*)** | 14142-14211 | 70 |  |  |  | 8 |
| ***nad5*** | 14214-15884 | 1671 | 556 | GTG | TAG | 2 |
| ***nad6*** | 15896-16375 | 480 | 159 | ATG | TAA | 11 |
| ***trnQ_2_*(*^CAA^*)** | 16409-16478 | 70 |  |  |  | 33 |
| ***nad3*** | 16484-16834 | 351 | 116 | ATG | TAA | 5 |
| ***trnL_1_*(*^CUN^*)** | 16867-16932 | 66 |  |  |  | 32 |
| ***trnF*** | 16971-17038 | 68 |  |  |  | 38 |
| ***trnA*** | 17055-17121 | 67 |  |  |  | 16 |
| ***nad1*** | 17126-18061 | 936 | 311 | ATG | TAA | 4 |
| ***nad4L*** | 18063-18345 | 283 | 94 | ATA | T- | 1 |
| ***trnW*** | 18346-18413 | 68 |  |  |  | 0 |

Table S1-6 Organization of the mitochondrial genome of *C. viginica*

| Gene | Position | Size  Nucleotide Amino acid | | Codon  Start Stop | | Intergenic  Nucleotides* |
| --- | --- | --- | --- | --- | --- | --- |
| ***cox1*** | 1-1623 | 1623 | 540 | ATG | TAA | 1 |
| ***rrnL*** 3’half | 1710-2430 | 721 |  |  |  | 86 |
| ***cox3*** | 2558-3429 | 872 | 290 | ATA | TA- | 127 |
| ***trnI*** | 3430-3495 | 66 |  |  |  | 0 |
| ***trnT*** | 3499-3567 | 69 |  |  |  | 3 |
| ***trnE*** | 3578-3646 | 69 |  |  |  | 10 |
| ***cob*** | 3647-4859 | 1213 | 403 | TTA | TAG | 0 |
| ***cox2*** | 4897-5589 | 693 | 230 | ATG | TAA | 37 |
| ***trnS_1_*(*^AGN^*)** | 5673-5743 | 71 |  |  |  | 83 |
| ***trnL_2_*(*^UUR^*)** | 5751-5817 | 67 |  |  |  | 7 |
| ***trnP*** | 5876-5944 | 69 |  |  |  | 58 |
| ***trnG*** | 6777-6841 | 65 |  |  |  | 832 |
| ***rrnS*** | 6842-7830 | 989 |  |  |  | 0 |
| ***trnM_1_*(*^ATG^*)** | 7831-7894 | 64 |  |  |  | 0 |
| ***trnK*** | 7923-7991 | 67 |  |  |  | 29 |
| ***trnC*** | 8008-8074 | 67 |  |  |  | 17 |
| ***trnV*** | 8097-8161 | 65 |  |  |  | 22 |
| ***trnD*** | 8179-8249 | 71 |  |  |  | 17 |
| ***rrnL*** 5’half | 8250-8997 | 748 |  |  |  | 0 |
| ***trnM_2_*(*^ATG^*)** | 9033-9098 | 66 |  |  |  | 35 |
| ***trnS_2_*(*^UCN^*)** | 9181-9254 | 74 |  |  |  | 82 |
| ***trnY*** | 9439-9513 | 75 |  |  |  | 184 |
| ***atp6*** | 9518-10192 | 675 | 224 | ATG | TAA | 4 |
| ***ND2*** | 10295-11290 | 996 | 331 | ATG | TAA | 102 |
| ***trnR*** | 11338-11403 | 66 |  |  |  | 47 |
| ***trnH*** | 11449-11513 | 65 |  |  |  | 45 |
| ***ND4*** | 11515-12864 | 1350 | 449 | ATG | TAA | 1 |
| ***trnN*** | 12919-12988 | 70 |  |  |  | 54 |
| ***ND5*** | 13126-14793 | 1668 | 555 | ATG | TAA | 137 |
| ***ND6*** | 14807-15268 | 462 | 153 | ATG | TAA | 13 |
| ***trnQ*** | 15278-15346 | 69 |  |  |  | 9 |
| ***ND3*** | 15352-15705 | 354 | 117 | ATG | TAA | 5 |
| ***trnL_1_*(*^CUN^*)** | 15726-15796 | 71 |  |  |  | 20 |
| ***trnF*** | 15804-15869 | 66 |  |  |  | 7 |
| ***trnA*** | 15892-15957 | 66 |  |  |  | 22 |
| ***ND1*** | 15959-16894 | 936 | 311 | ATG | TAA | 1 |
| ***ND4L*** | 16897-17176 | 280 | 93 | ATG | T- | 2 |
| ***trnW*** | 17177-17243 | 67 |  |  |  | 0 |

* Numbers correspond to the nucleotides separating different genes.

“-” indicates termination codons completed via polyadenylation.
