# Supplementary material for: Unusual conservation of mitochondrial gene order in Crassostrea oysters: evidence for recent speciation in Asia
Source: BMC Evol Biol. 2010 Dec 28;10:394. doi: 10.1186/1471-2148-10-394 (PMC3040558; doi:10.1186/1471-2148-10-394)

**Figure S1 Comparison of the potential secondary structures of the 25 inferred tRNAs among five Crassostrea oyster mtDNAs.**

Codons recognized are shown for the pairs of Leucine and Serine. The two tRNA duplications of Methionine, Lysine and Glutamine are named *trnM1*, *trnM2*, *trnK1*, *trnK2*, *trnQ1* and *trnQ2*, respectively. The tRNA secondary structure of *C. gigas* is referred to as the standard structures. The shade nucleotides indicate the changed nucleotides. The shade and the arrows denote the base deletion and insertion relative to the tRNA structures of *C. gigas*.


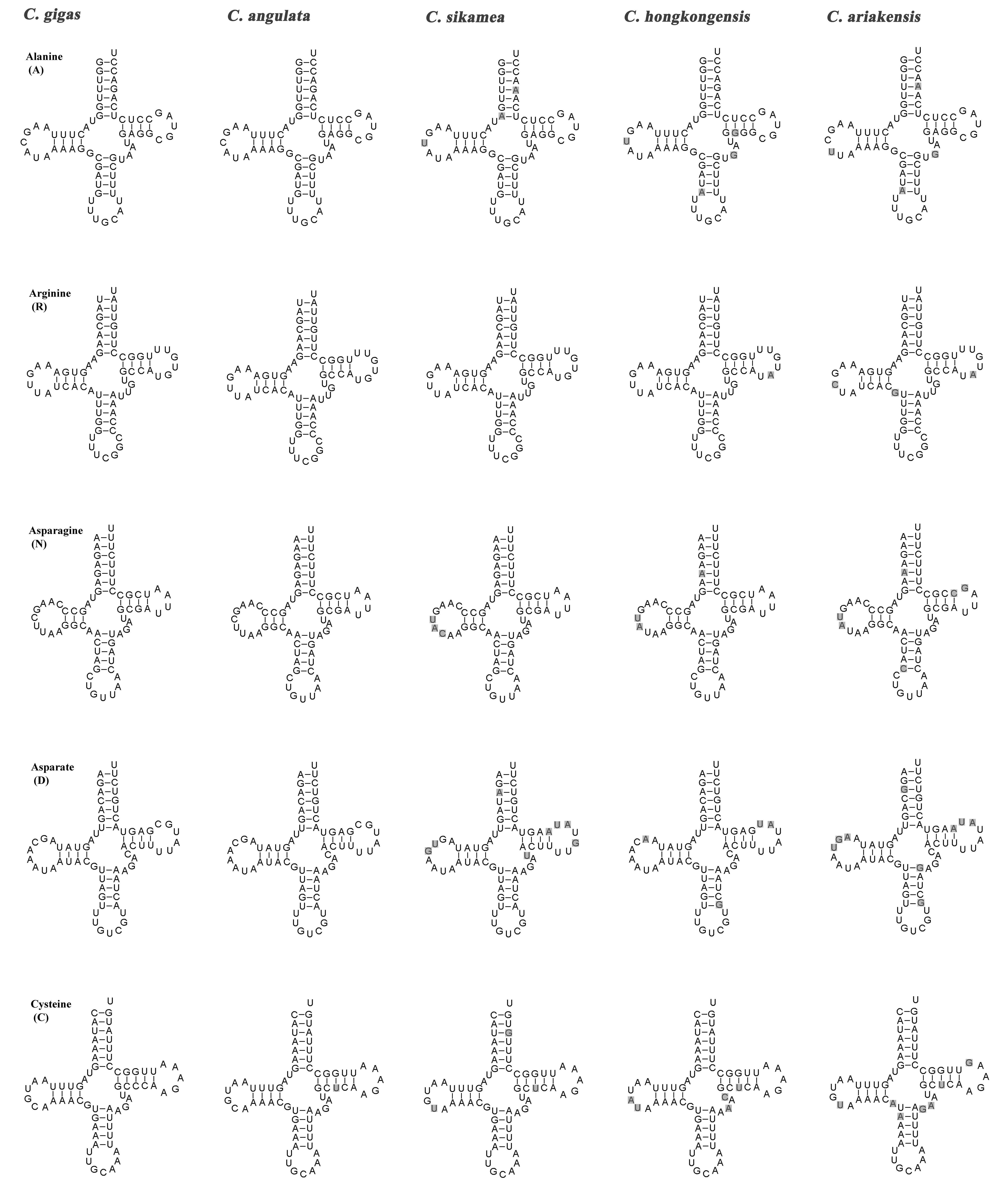


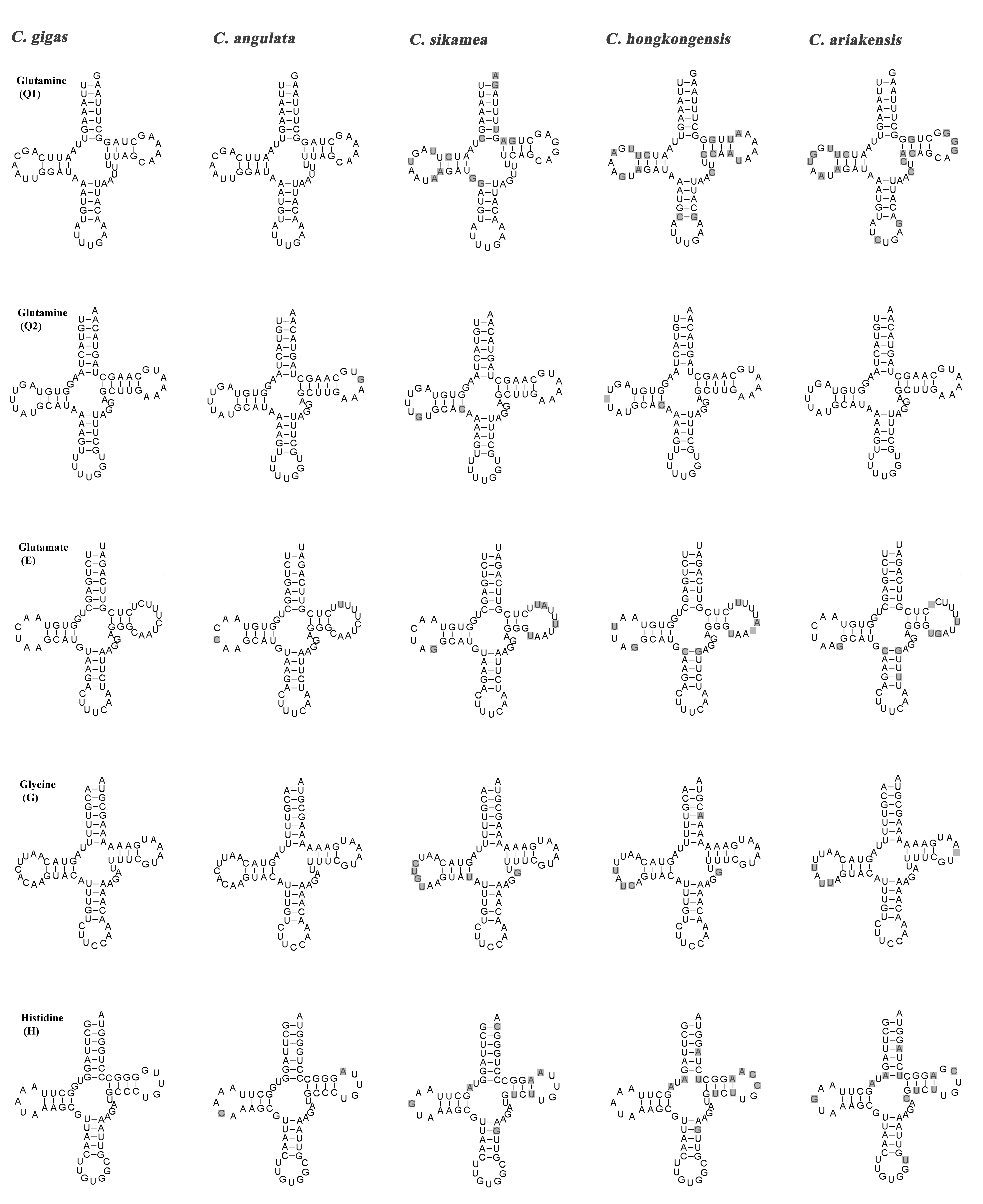

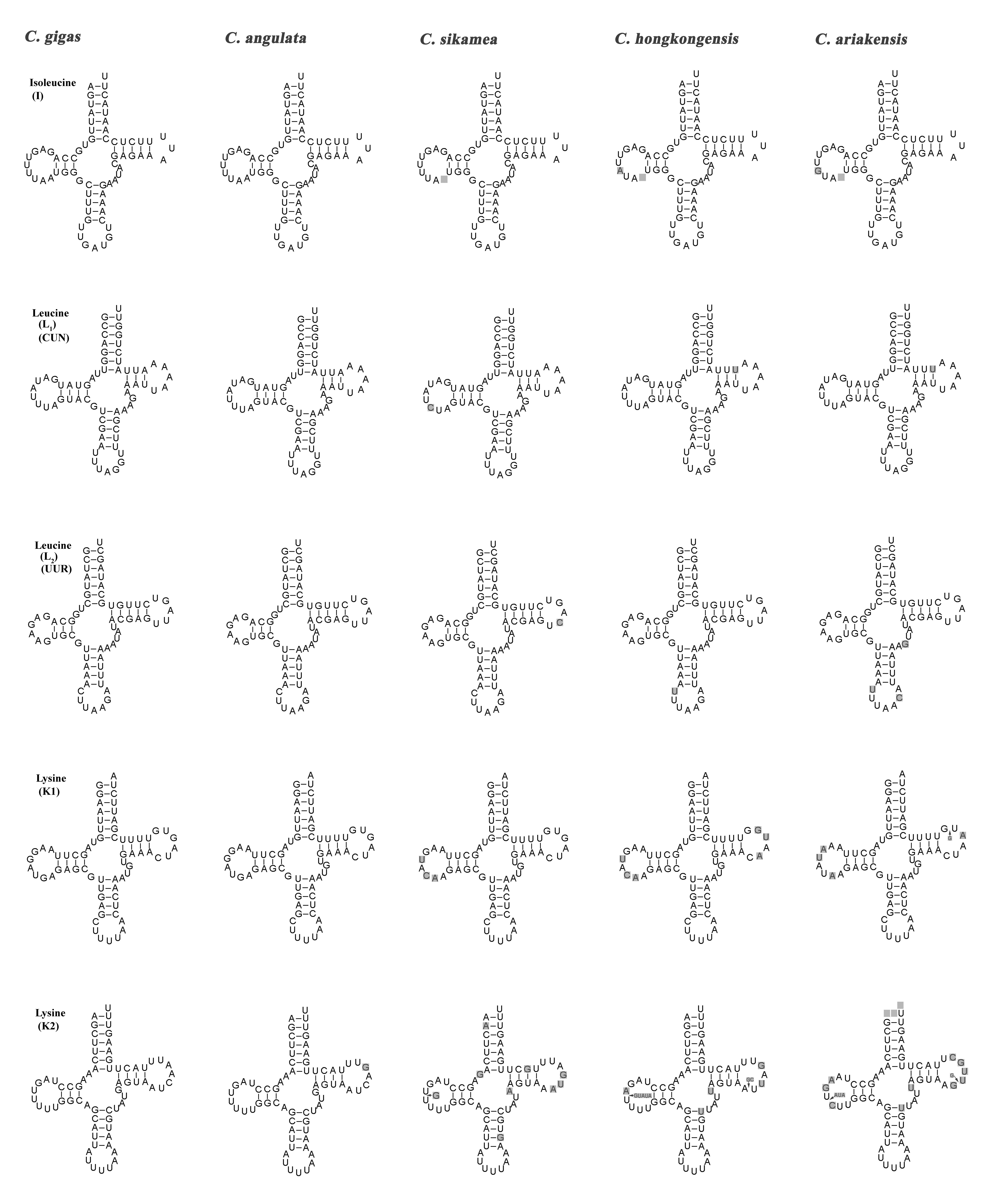

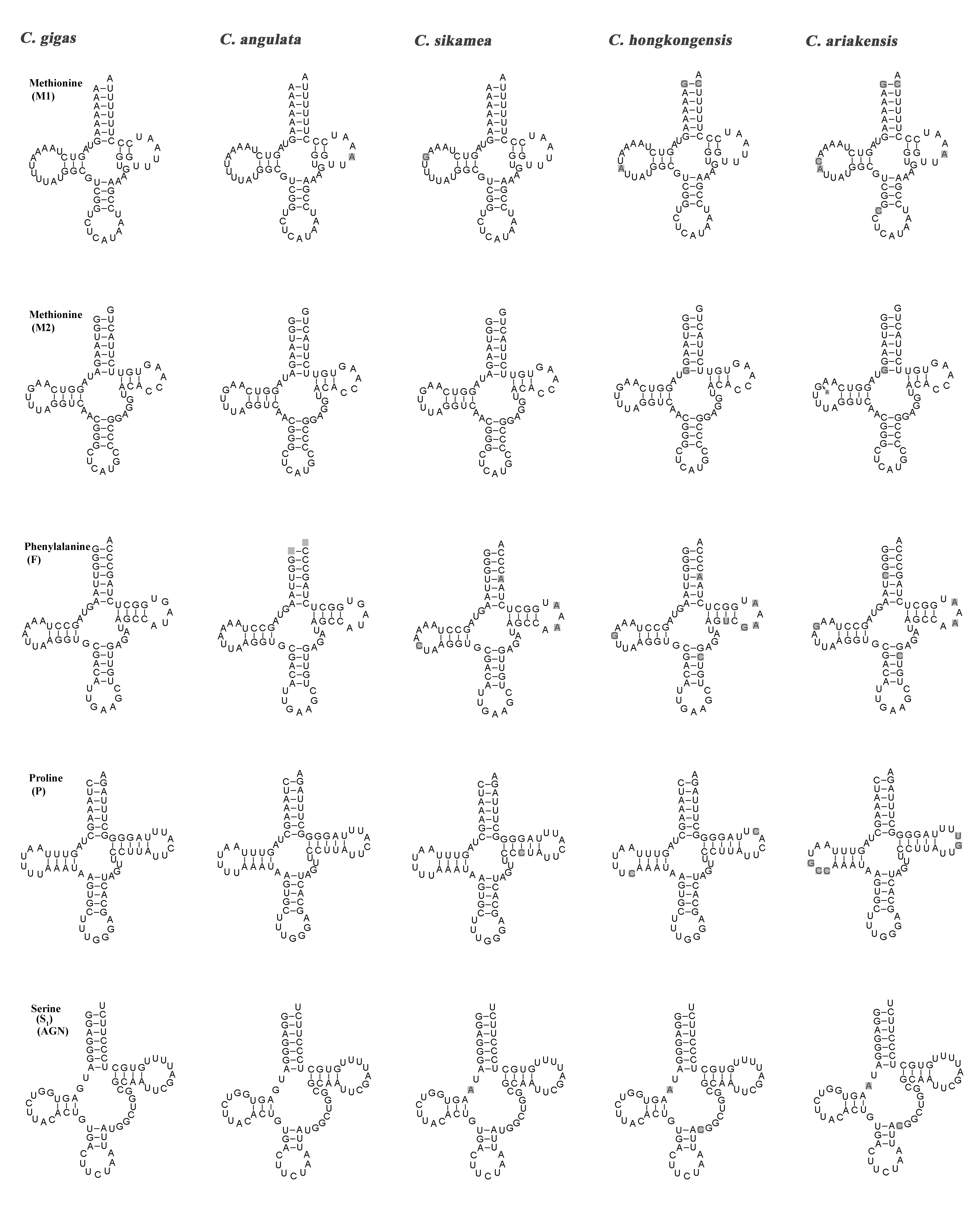

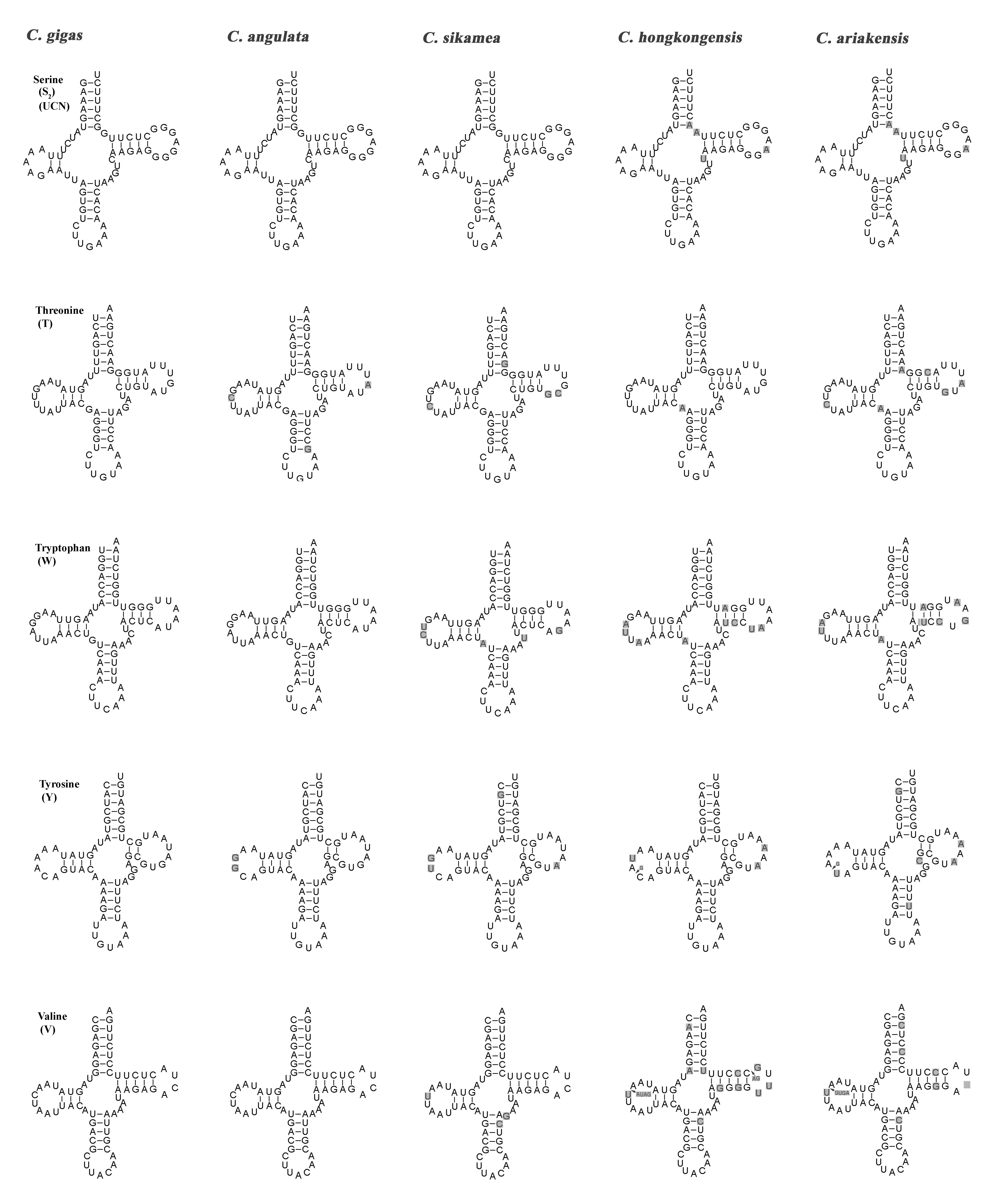

Supplement: Additional file 3 — Figure S1: Comparison of the potential secondary structures of the 25 inferred tRNAs among five Crassostrea oyster mtDNAs. [file 1471-2148-10-394-S3.DOC]
