# Supplementary material for: Unusual conservation of mitochondrial gene order in Crassostrea oysters: evidence for recent speciation in Asia
Source: BMC Evol Biol. 2010 Dec 28;10:394. doi: 10.1186/1471-2148-10-394 (PMC3040558; doi:10.1186/1471-2148-10-394)
Supplement: Additional file 4 — Table S3: Major primers used in amplifying the mitochondrial genomes. [file 1471-2148-10-394-S4.DOC]

Table S3 Major Primers used in amplifying the mitochondrial genomes

| **Name** | **Sequence** | **Length** | **Reference** |
| --- | --- | --- | --- |
| *LCO*1490 | GGTCAACAAATCATAAAGATATTGG | 25 | Folmer et al., 1994 |
| *HCO*2198 | TAAACTTCAGGGTGACCAAAAAATCA | 26 |  |
|  |  |  |  |
| *cox2*F1 | AAGCWAATWGGNCATCARTGRTATTG | 26 | Burger et al., 2007 |
| *cox2*R1 | CTCCRCATATTTCNGARCATTGNCC | 29 |  |
|  |  |  |  |
| CG16406F | TTGGGTTCTTAGGGTTTATTGTCT | 24 | This study |
| CG2956R | GCCAGGCATTTCTACTTTAACACT | 24 |  |
|  |  |  |  |
| CG2938F | TAAAGTAGAAATGCCTGGCGTGTT | 24 |  |
| CG7505R | CATAAGTAGCGGGAAGTAAGCAAA | 24 |  |
|  |  |  |  |
| CG7272F | GGGCGTAAGGGAAGGAAGAAGG | 22 |  |
| CG12349R | TTAGAAGCGTACCCAGCAGTCCCT | 24 |  |
|  |  |  |  |
| CG12328F | GGACTGCTGGGTACGCTTCTA | 22 |  |
| CG16431R | CCAGACAATAAACCCTAAGAACCC | 24 |  |
|  |  |  |  |
| HK-*cox1*-*cox2*-F | TCCACCCTTATCCACTTACTC | 21 |  |
| HK-*cox1*-*cox2*-R | TTTCAACTCATCTTCAGGCAC | 21 |  |
|  |  |  |  |
| HK-*cox2*-*nad5*-F | ATAGTGCCTGAAGATGAGTTGAA | 23 |  |
| HK-*cox2*-*nad5*-R | AAAGACGCTACTAACTCACCACCA | 24 |  |
|  |  |  |  |
| HK-*nad5*-*cox1*-F | CCTCTTTGCCTGCTGCTGTATT | 22 |  |
| HK-*nad5*-*cox1*-R | GGAGTAAGTGGATAAGGGTGGA | 22 |  |
